# Force-responsive symmetric cell divisions orient stomata along global tissue axes

**DOI:** 10.1073/pnas.2529768123

**Published:** 2026-06-30

**Authors:** Kensington S. Hartman, Bianca Y. Lopez, Juan H. Gonzalez, Madison E. Goetz, Aviel Cleveland, Andrew Muroyama

**Affiliations:** ^a^ Department of Cell and Developmental Biology, Division of Biological Sciences, University of California San Diego, La Jolla, CA 92093

**Keywords:** plant development, stomata, cell division, polarity

## Abstract

Stomata, microscopic pores that regulate gas exchange in plants, are patterned according to conserved pathways that regulate their physiology. Here, we identify a mode of stomatal patterning that depends on previously unrecognized regulation of the final symmetric cell division (SCD) that creates paired guard cells. SCDs are aligned by tensile stress at both the subcellular and supracellular scales, creating a globally polarized stomatal field that tracks the major axes of tissue growth. By identifying KATANIN 1 as a critical regulator of symmetric division orientation, we show that stress-based division orientation is required to prepattern stomatal morphology and pore creation. We find that expansion of neighboring cells nonautonomously controls symmetric division orientation, linking stomatal alignment to overall leaf shape. Finally, we show that polarized stomatal fields are widespread across plant genera and their species-specific alignment patterns are consistent with the force-based mechanism we identify in *Arabidopsis*. This force-responsive pathway provides a unifying model that explains long-standing observations of stomatal organization across species.

## Significance

Plant development requires the precise control of cell division, growth, and fate transitions. How these are coordinated to generate developmentally conserved patterns remains mysterious for many plant tissues. We identified a pathway that integrates mechanical information to orient the final symmetric cell division during the formation of stomata, microscopic pores that are essential for gas exchange. This force-responsive mechanism generates a polarized stomatal field across the surface of aerial organs and is required for pore formation, highlighting a function for mechanics during leaf development.

Plant morphogenesis relies on oriented cell divisions to generate cellular diversity and control tissue architecture. These divisions can be classified as either symmetric or asymmetric depending on whether they generate daughter cells with equivalent or distinct identities, respectively. Precise spatiotemporal control of both asymmetric cell division (ACD) and symmetric cell division (SCD) is essential to establish the stereotyped cellular patterns that define and regulate the physiology of various plant organs (1, 2). Hormone gradients (3–5), niche-derived soluble signals (6), mobile transcription factors (7, 8), and cell-autonomous patterning proteins (9, 10) have all been shown to regulate proliferative potential and fate acquisition. In addition to these well-established pathways, tissue mechanics are increasingly appreciated as critical drivers of organ formation (11, 12). For example, forces derived from organ and cell shape inform division patterns in the shoot apical meristem, where divisions within relatively homogenous cell populations of stereotyped geometry are oriented to minimize tensile stress (13). However, how mechanics instruct the development of other plant tissues remains an important and open question.

The leaf epidermis is a classic example of a tissue whose cellular pattern is established by pathways that coordinate oriented cell divisions with cell-fate switches (14). During leaf development, the stomatal lineage generates most of the cells and structures in the epidermis, including trichomes, pavement cells and stomata, microscopic pores formed between paired guard cells (GCs). In the early stages of the lineage in *Arabidopsis thaliana*, meristemoids perform rounds of ACD that modulate the ratio of stomata to pavement cells in the leaf. Once the meristemoid stops dividing asymmetrically and becomes a guard mother cell (GMC), it performs a final SCD to create the paired GCs that form a stomate.

Despite significant variation in cell and leaf morphology, conserved stomatal patterns can be observed across different species. The most well-characterized of these is the “one-cell spacing rule,” which depends on oriented ACDs and local inhibition of stomatal identity via small peptide signaling (15–20). Other stomatal patterns have been anecdotally noted within certain plant families (21, 22). We were particularly struck by the robust alignment of stomata along the proximodistal axis of grass leaves, which suggested that uncharacterized mechanisms control SCD orientation, at least in some species (23). Division plane defects in GMCs have been noted in *Zea mays* (24), *Oryza sativa* (25), and *Brachypodium distachyon* (26) mutants harboring loss-of-function alleles of MUTE orthologs, which specify GMC identity and subsidiary cell recruitment. However, the mechanistic basis underlying this SCD control in these or other plant species has not been systematically explored.

## Results

### Symmetric Divisions That Create Stomata Are Aligned in the *Arabidopsis* Leaf Epidermis.

**Fig. 1.**
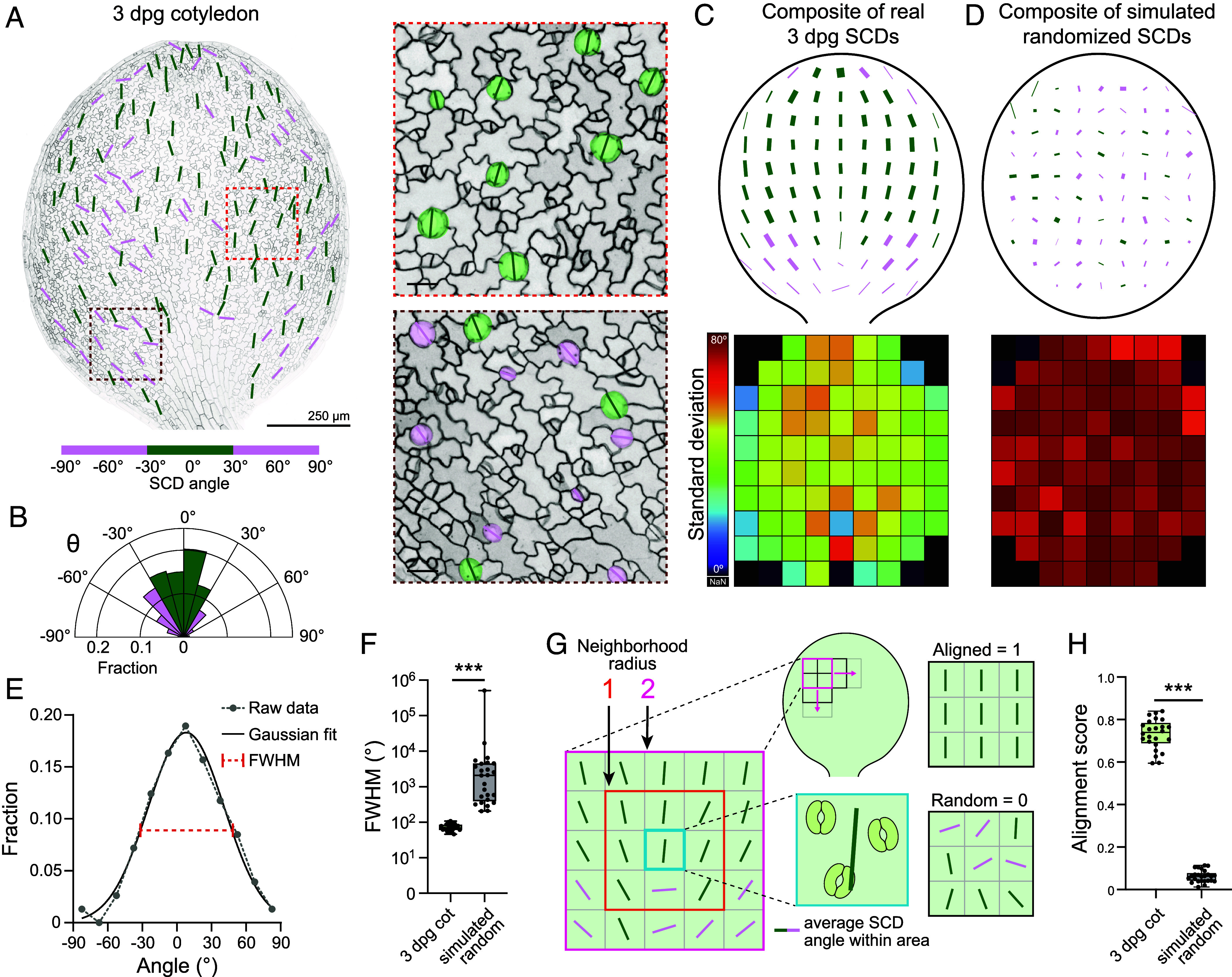
SCDs in GMCs are aligned in the *Arabidopsis* leaf epidermis. (*A*) Representative image of a 3 dpg ML1p::mCherry-RCI2A-expressing cotyledon (*Left*) and zoomed regions (*Right*) with the SCDs color-coded according to their orientation relative to the proximodistal axis. [Scale bar, 250 µm (*Left*) and 25 µm (*Right*).] (*B*) Polar histogram of SCD angles relative to the cotyledon’s proximodistal axis (0°). (*C*) (*Top*) Composite 3 dpg cotyledon generated from experimentally derived data. (*Bottom*) Associated map of the corresponding angular deviations. n = 24 cotyledons, 81 to 221 stomata/cotyledon. (*D*) (*Top*) Composite “cotyledon” from simulated cotyledons with randomly oriented SCDs. (*Bottom*) Associated map of the corresponding angular deviations. n = 25 cotyledons, 150 stomata/cotyledon. (*E*) The distribution of SCD angles from the example in (*A*) and associated Gaussian fit and FWHM value. (*F*) FWHM values of the 3 dpg and simulated, randomized cotyledons from (*B* and *C*), respectively. (*G*) Alignment by Fourier Transform (AFT) method for calculating local alignment among neighboring stomata. (*H*) Alignment scores for the 3 dpg and simulated, randomized cotyledons from (*B* and *C*), respectively.

Previous qualitative observations of small regions of fully developed leaves led to the conclusion that the SCDs that generate stomata are randomly oriented in *Arabidopsis* (27). We revisited this assumption by mapping the SCD landscape across the leaf epidermis, which can be derived from the placement of the cell walls separating paired GCs (*SI Appendix*, Fig. S1*A*). For consistency, we measured SCD orientation from GC pairs with a pore. We confirmed that this method faithfully reports SCD orientation, even following cell expansion (*SI Appendix*, Fig. S1*B*). Annotating all SCDs across the entire abaxial epidermis of 3 days postgermination (3 dpg) cotyledons made it visually apparent that, contrary to previous assumptions, division orientation is strongly biased along the cotyledon’s proximodistal axis (Fig. 1 *A*–*C*). Throughout, we refer to this global division orientation bias as “stomatal alignment.”

If SCD orientation were random, with stomatal angles uniformly distributed between −90° and 90° around the proximodistal axis (0°), the average orientation of the stomatal field would vary drastically across the leaf, with short mean resultant vectors at any given position within the field. We verified this prediction by simulating cotyledons with SCD orientations drawn from a uniform random distribution (Fig. 1*D* and *SI Appendix*, Fig. S1*C*). In contrast, we found the average stomatal orientation of our 3 dpg cotyledons was biased along the proximodistal axis and the mean resultant vectors were much longer (Fig. 1*C*). To quantitatively investigate whether SCD orientation in 3 dpg cotyledons deviates from random simulations, we developed measures to compare three parameters of stomatal alignment. i) The between-sample variability of SCD orientation at a given position within the tissue can be assessed by calculating the SD of SCD angles at defined intervals across the leaf epidermis. SCD angle was highly variable between the simulated randomized samples, as expected, while variability was low between real 3 dpg cotyledons (Fig. 1 *C* and *D*). ii) Global SCD alignment to the proximodistal axis can be measured by fitting the distribution of SCD angles within a single sample with a Gaussian function and calculating the full-width half-maximum (FWHM) value; smaller and larger FWHM values represent higher and lower alignment, respectively (Fig. 1*E*). Stomata were consistently aligned with the proximodistal axis in 3 dpg cotyledons but FWHM values were dramatically larger in simulated samples, as would be expected when the distribution of SCD angles approaches random (Fig. 1*F*). iii) We measured local concordance of SCD orientation among neighboring stomata and found that SCDs showed strong local alignment in 3 dpg cotyledons but very poor alignment in our randomized samples (Fig. 1 *G* and *H*). Therefore, by all three metrics, stomatal alignment in 3 dpg *Arabidopsis* cotyledons is nonrandom and strongly biased along the proximodistal axis.

Next, we evaluated if global SCD orientation changes over development by quantifying stomatal alignment in 3 to 7 dpg cotyledons, over which time there is an ~fourfold increase in the number of stomata per cotyledon. Stomatal alignment to the proximodistal axis and alignment between neighboring stomata both decreased from 3 to 5 dpg, although they always remained biased in the proximodistal direction (*SI Appendix*, Fig. S1 *D*–*G*). Finally, we checked whether stomatal alignment is specific to cotyledons or is found in other stomata-containing aerial tissues. True leaves also showed global stomatal alignment biased along the proximodistal axis and SCDs were almost universally oriented along the proximodistal axis in sepals (*SI Appendix*, Fig. S1 *H*–*L*). From these data, we conclude that division orientation in GMCs is actively regulated across *Arabidopsis* tissues, providing an experimental system to explore the mechanisms controlling polarized SCDs during plant morphogenesis.

### Key Regulators of Polarized Division and Cell Fate in the Stomatal Lineage Do Not Control SCD Orientation.

**Fig. 2.**
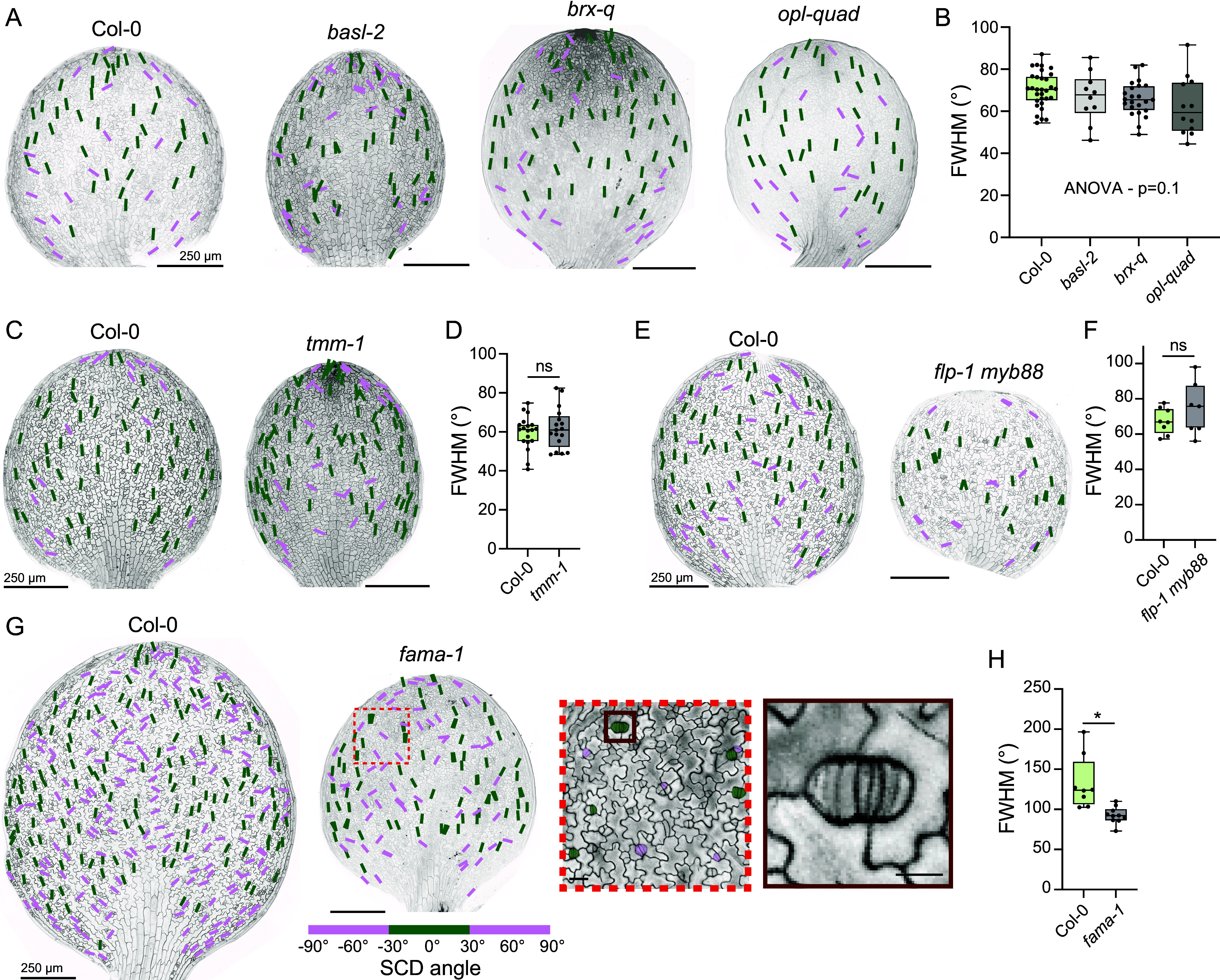
SCD orientation is not controlled by key stomatal lineage regulators. (*A*) 3 dpg cotyledons from the indicated genotypes with color-coded SCDs. (Scale bar, 250 µm.) (*B*) FWHM values for Col-0, *basl-2*, *brx-q*, and *opl-quad*. n = 31 (Col-0, 51 to 158 stomata/cotyledon), 10 (*basl-2*, 35 to 139 stomata/cotyledon), 23 (*brx-q*, 71 to 199 stomata/cotyledon), and 12 (*opl-quad*, 60 to 94 stomata/cotyledon*)* cotyledons. (*C*) 3 dpg Col-0 and *tmm-1* cotyledons with color-coded SCDs. (Scale bar, 250 µm.) (*D*) FWHM values for Col-0 and *tmm-1*. n = 19 (Col-0, 52 to 126 stomata/cotyledon) and 17 (*tmm-1*, 54 to 236 stomata/cotyledon) cotyledons. (*E*) 3 dpg Col-0 and *flp-1 myb88* cotyledons with color-coded SCDs. (Scale bar, 250 µm.) (*F*) FWHM values for Col-0 and *flp-1 myb88*. n = 8 (Col-0, 55- to 138 stomata/cotyledon) and 7 (*flp-1 myb88* 27 to 49 stomata/cotyledon) cotyledons. (*G*) 5 dpg Col-0 and *fama-1* cotyledons (*Left*) and zoomed regions showing parallel, oriented SCDs in *fama-1* (*Right*) with color-coded SCDs. [Scale bar, 250 µm (two *Leftmost*), 25 µm (*Middle*), and 10 µm (*Right*).] (*H*) FWHM values for Col-0 and *fama-1*. n = 8 (Col-0, 249 to 413 stomata/cotyledon) and 10 (*fama-1*, 89 to 183 stomata/cotyledon) cotyledons.

To determine how GMC SCDs are oriented, we started by testing if known regulators of cell division in the stomatal lineage are required to generate the polarized stomatal field. ACDs in the *Arabidopsis* stomatal lineage are oriented cell-autonomously by the plasma membrane-associated polar proteins BREAKING OF ASYMMETRY IN THE STOMATAL LINEAGE (BASL) and members of the BREVIS-RADIX family (BRXf) (9, 28). Even though fluorescent BASL and BRXf reporters can be planar polarized along global leaf axes (29–31) and our reporters showed persistent, albeit nonpolar, expression in GMCs (*SI Appendix*, Fig. S2*A*), we found that SCD orientation was unchanged in *basl-2* and *brx-q*, a quadruple, loss-of-function mutant in four of the five BRXf genes (Fig. 2 *A* and *B*). The OCTOPUS-LIKE (OPL) protein family was recently shown to polarize opposite to BASL and BRXf in asymmetrically dividing cells and, intriguingly, OPL2 localized to the shared cell wall separating GCs immediately after division (32). We found that SCDs continued to be oriented along the proximodistal axis in the quadruple *opl1-1 opl2-3 opl3-1 opl4-1* mutant (*opl-quad*), indicating that the polar proteins controlling ACD and division potential are not required for SCD orientation (Fig. 2 *A* and *B*).

Global SCD alignment was unchanged in a *TOO MANY MOUTHS* loss-of-function mutant (*tmm-1*) and a *FOUR LIPS* (*FLP*) *MYB88* mutant (*flp-1 myb88*) (33, 34), demonstrating that one-cell spacing is not required for oriented SCDs (Fig. 2 *C*–*F*). *FAMA* expression is initiated in late-stage GMCs before they undergo SCD and is required to both limit GMC divisions and establish proper GCs identity (35). Surprisingly, even though overall cotyledon growth was slightly suppressed in *fama-1*, GMC divisions continued to be aligned with the proximodistal axis in *fama-1* at a frequency comparable to that seen in wild-type (WT) cotyledons of similar size (Fig. 2 *G* and *H*). Therefore, neither known regulators of stomatal lineage ACD nor transcription factors controlling GMC division and GC fate are necessary for oriented GMC SCDs.

### GMC Division Orientation Is Not Controlled by a Vein-Secreted Signal.

Because the SCD field closely mirrored the organization of the underlying vasculature and a tight relationship between stomatal distribution and veins has been noted in some species (36–38), including grasses (22, 39), we tested whether division orientation in GMCs depended on proper vein specification. To do this, we measured SCD orientation in four mutants, *tir1-1 afb2-3 afb3-4* (40), *scarface* (*scf-9*) (41), *dot3-*2 and *dot5-2* (42), that have defects in vein development. SCD orientation across the leaf epidermis was unaffected in all four of these mutant backgrounds, leading us to conclude that GMC division orientation does not depend on the prepatterned secretion of a vein-derived mobile signal (*SI Appendix*, Fig. S2 *B*–*G*).

### Auxin Does Not Drive Polarized SCD Orientation.

Auxin gradients control cell division patterns in many developing plant tissues (43, 44), but the role of auxin in the final SCD has not been characterized. We tested whether auxin-mediated signaling controls GMC division orientation by measuring stomatal alignment in seedlings grown on plates containing the auxin indole-3-acetic acid (IAA) or *N*-1-naphthylphthalamic acid (NPA), an inhibitor of the PIN auxin efflux transporters (45). Despite causing changes in overall cotyledon size (*SI Appendix*, Fig. S2 *H* and *J*), neither treatment negatively disrupted GMC division orientation in 3 dpg seedlings (*SI Appendix*, Fig. S2 *H*–*K*). We noted a slight increase in stomatal alignment in our seedlings exposed to 1 µM IAA, but as the FWHM values were comparable to those observed in Col-0 seedlings with similar stomatal counts, we believe that this change reflects an IAA-induced delay in cotyledon development rather than a specific effect on SCD orientation. Together with the observation that SCD orientation is not altered in *tir1-1 afb2-3 afb3-4* (*SI Appendix*, Fig. S2 *F* and *G*), we conclude that auxin is not a critical regulator of polarized SCDs in the stomatal lineage.

### GMC Morphology Predicts Division Orientation.

**Fig. 3.**
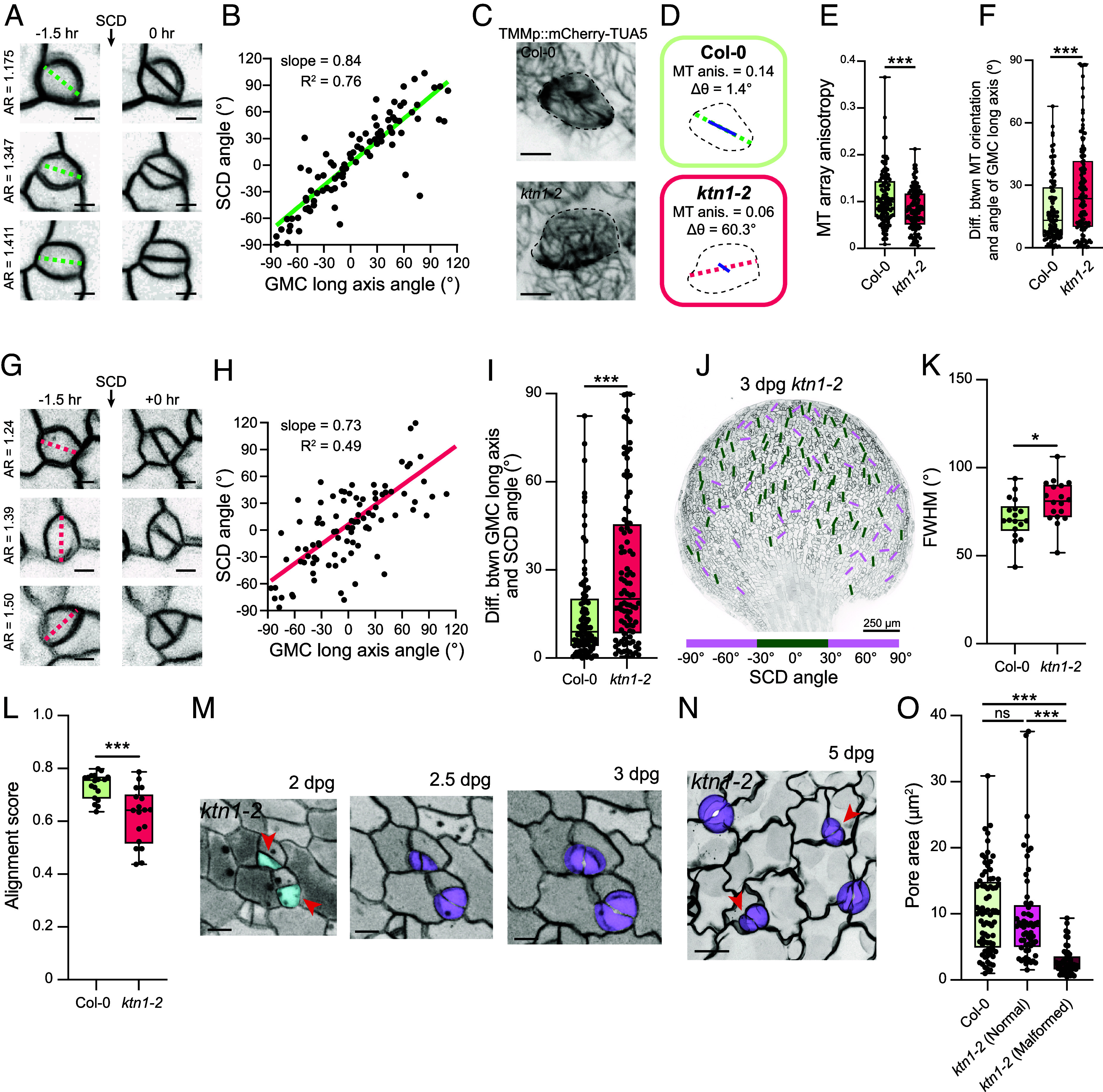
SCD orientation follows GMC morphology and requires KATANIN 1. (*A*) GMC divisions from 3 dpg ML1p::mCherry-RCI2A-expressing cotyledons (dashed green line—GMC long axis). (Scale bar, 5 µm.) (*B*) The angle of the GMC long axis immediately before mitosis relative to the SCD angle (green line—linear fit). n = 91 cells. (*C*) Microtubule organization in TMMp::mCherry-TUA5-expressing Col-0 and *ktn1-2* GMCs. (Scale bar, 5 µm.) (*D*) Simplified representation of the GMCs in *C* showing the cell outline (thin dashed line), the GMC long axis (dashed green or magenta line), and the major direction of microtubule alignment (solid blue line). (*E*) Anisotropy of the microtubule network in Col-0 and *ktn1-2* GMCs. n = 125 (Col-0) and 124 (*ktn1-2*) cells. (*F*) Differences between the orientation of the microtubule array and the GMC long axis. n = 103 (Col-0) and 124 cells (*ktn1-2*) cells. (*G*) Abnormally oriented SCDs in *ktn1-2* (dashed magenta line—GMC long axis). (Scale bar, 5 µm.) (*H*) The angle of the *ktn1-2* GMC long axis immediately before mitosis relative to the SCD angle (magenta line—linear fit). n = 96 cells. (*I*) Differences between the GMC long axis angle and the angle of the SCD. n = 91 (Col-0) and 96 cells (*ktn1-2*) cells. (*J*) 3 dpg *ktn1-2* cotyledon with color-coded SCDs. (Scale bar, 250 µm.) (*K*) FWHM values for 3 dpg Col-0 and *ktn1-2* cotyledons. n = 18 (Col-0, 77 to 177 stomata/cotyledon) and 18 (*ktn1-2*, 38-224) cotyledons. (*L*) Alignment scores for Col-0 and *ktn1-2* cotyledons. n = same as *K*. (*M*) Misoriented *ktn1-2* SCDs tracked over 24 h. (Scale bar, 10 µm.) (*N*) 5 dpg ML1p::mCherry-RCI2A-expressing *ktn1-2* cotyledon with malformed stomata (red arrows). (Scale bar, 25 µm.) (*O*) Pore areas in 5 dpg Col-0 and *ktn1-2* cotyledons. n = 76 (Col-0), 57 (normal, *ktn1-2*), and 50 (malformed, *ktn1-2*) pores.

Several models have been developed to describe how cell division can follow morphological or mechanical cues in the absence of other extrinsic or intrinsic signals (13, 46), and anecdotal descriptions of GMC behavior suggested that they may divide along their long axes (47, 48). To systematically examine whether GMC morphology robustly predicts SCD orientation, we performed time-lapse imaging in seedlings expressing an epidermal-specific plasma membrane marker (ML1p::mCherry-RCI2A) that allowed us to monitor GMC growth and associated division patterns. In our time-lapse movies, GMC morphology and division orientation were strongly correlated, with the newly created cell wall closely matching the GMC’s long axis (R^2^ = 0.76) (Fig. 3 *A* and *B*), suggesting that cell shape might be an important determinant of SCD orientation in the stomatal lineage.

WT GMCs expand before undergoing SCD, approaching circularity before mitotic entry (median circularity = 0.907). This raised the possibility that the nearly isotropic GMC shape would introduce some degree of stochasticity in division plane selection, similar to what has been seen in other cases (49). Indeed, SCD angles in Col-0 better matched the GMC long axis in more anisotropic GMCs, and SCDs in highly isotropic GMCs deviated most significantly from the GMC long axis (*SI Appendix*, Fig. S3 *A* and *B*). To test whether increasing GMC anisotropy is sufficient to increase the predictive power of GMC morphology, we examined SCD orientation in the *TRM678* mutant, which forms abnormally shaped GMCs (50, 51) (*SI Appendix*, Fig. S3*C*). Based on time-lapse imaging in *trm678* ML1p::mCherry-RCI2A seedlings, we found that increasing GMC anisotropy significantly improved concordance between the GMC long axis and the SCD angle (R^2^ = 0.95) (*SI Appendix*, Fig. S3 *D*–*F*). From these data, we conclude that GMCs divide along their long axes and that their near circularity before mitotic onset in Col-0 introduces some stochasticity in division plane selection during WT GMC divisions.

### KTN1 Controls Microtubule Organization and Division Orientation in GMCs.

In other plant tissues, cells that divide along their long axes do so because they follow the direction of greatest tensile stress (13). No work has yet identified a role for mechanical forces in controlling division orientation during stomatal patterning, but our time-lapse data suggest that force-based cues might be the dominant regulator of division orientation in *Arabidopsis* GMCs.

The magnitude and direction of the stresses within individual plant cells are difficult to experimentally determine, but cortical microtubules are generally considered to be force-responsive in plants and will typically align parallel to the direction of greatest stress (52). Using a stomatal lineage-specific microtubule reporter line (TMMp::mCherry-TUA5), we quantified microtubule anisotropy and alignment on the periclinal surface of GMCs and found that microtubules were well aligned along the GMC long axis (Fig. 3 *C*–*F*). Because microtubule ordering along the axis of greatest stress is dependent on the microtubule-severing protein KATANIN 1 (KTN1) in the shoot apical meristem (53) and cortical microtubules were less anisotropic in young *ktn1* leaves (54), we quantified microtubule organization in *ktn1* GMCs by introgressing the TMMp::mCherry-TUA5 reporter into *ktn1-2*. Microtubule network anisotropy and alignment along the GMC long axis were both decreased upon *KTN1* loss (Fig. 3 *C*–*F*), indicating that the GMC long axis is indeed its axis of maximum stress.

*ktn1* GMCs are slightly more anisotropic than those in Col-0 (*SI Appendix*, Fig. S3*G*), so we were surprised to find increased randomization of SCD orientation in *ktn1-2*, with many SCDs that bisected the GMC along a shorter path (Fig. 3 *G*–*I*). Accordingly, global SCD alignment along the proximodistal axis was significantly decreased in *ktn1-2*, as was the alignment between neighboring stomata (Fig. 3 *J*–*L*). Notably, the striking variability in local alignment between *ktn1-2* cotyledons indicated that the decreased FWHM values in *ktn1-2* are not due to a lateral reorientation of SCDs but rather a significant increase in SCD randomization (Fig. 3*L*). To confirm that KTN1 is required for division orientation in GMCs, we measured SCD angles in two additional *KTN1* alleles, *lue1* (55) and *ktn1-20* (56), and found a decrease in global alignment in both (*SI Appendix*, Fig. S3 *H* and *I*). Taken together, these data are consistent with a model where GMC divisions are oriented along the axis of greatest stress, which both 1) informs and is read out by the major axis of the cortical microtubule array and 2) coincides with the long axis in Col-0 GMCs.

The SCD randomization in *ktn1-2* presented an opportunity to test whether defects in SCD orientation have phenotypic consequences on stomatal formation. By monitoring epidermal development from 2 to 3 dpg in *ktn1-2* cotyledons, we found that GCs that were created from SCDs that failed to align along the GMC long axis exhibited striking morphological defects during guard cell maturation (Fig. 3*M*). By 5 dpg, the *ktn1-2* epidermis contained two distinct classes of stomata: those with kidney-shaped GCs that were morphologically indistinguishable from Col-0 stomata and those that were misshapen, with increased lateral expansion and reduced expansion along the shared wall (Fig. 3*N*). We observed cases of similar misshapen stomata in both *lue1* and *ktn1-*20 (*SI Appendix*, Fig. S3*J*). Cortical microtubules were organized in the characteristic radial arrays that define interphase microtubule organization in GCs in both normal and misshapen stomata, indicating that malformed stomata in *ktn1-2* are not caused by gross differences in microtubule organization post division (*SI Appendix*, Fig. S3*K*). Notably, pore area was significantly decreased in malformed stomata in *ktn1-2* but was unaffected in normal *ktn1-2* stomata, which had identical pore areas as those formed in Col-0 (Fig. 3*O* and *SI Appendix*, Fig. S3*L*). These results identify a previously unrecognized function for oriented SCD in controlling GC morphology and pore formation.

### Developmentally Regulated Growth Patterns Globally Align GMC SCDs.

Our time-lapse data demonstrated that GMC morphology cell autonomously predicts SCD orientation of any individual cell but does not, on its own, explain why SCDs are oriented in a polarized field across the tissue. Indeed, while concordance between the GMC long axis and division plane is increased in *trm678*, global alignment is actually reduced relative to Col-0 cotyledons (*SI Appendix*, Fig. S3 *M* and *N*). While analyzing GMC behavior and SCD orientation in WT and stomatal patterning mutants, we made several observations that hinted that anisotropic GMC expansion might align neighboring stomata to generate the polarized SCD field across the epidermis.

(1) We observed regional differences in stomatal alignment, notably SCDs near the petiole that were locally aligned and fanned outward along the leaf margins (Fig. 1*A*), leading to a decrease in local stomatal alignment over distance (*SI Appendix*, Fig. S4*A*). These differences were magnified in other places, including the serrations of true leaves (*SI Appendix*, Fig. S4*B*).
(2) Stomatal alignment differed significantly between the 3 dpg abaxial and adaxial surfaces (*SI Appendix*, Fig. S4*C*), which modeling suggests principally grow in different directions to generate the cup-shaped cotyledon (57, 58).
(3) Stomatal clusters can arise from GMC divisions that occur at the same time (synchronous) or at distinct developmental stages (asynchronous). Closer examination of the paired stomata in *basl-2* and *flp-1 myb88* revealed that synchronous pairs were well aligned with each other while asynchronous ones were significantly less well-aligned (*SI Appendix*, Fig. S4 *D* and *E*). This observation suggested that the GMC position alone is not sufficient to predict GMC orientation. Instead, SCD orientation depends on the interaction between GMC position and developmental stage.
(4) While screening for mutants with altered SCD orientation, we identified two, *kan1-11 kan2-5* (59) and *rot3-1* (60), where leaf shape and stomatal alignment were both changed. Stomatal alignment along the proximodistal axis was decreased in the squat *rot3-1* leaves and was hyperaligned in the elongated 10 dpg *kan1-11 kan2-5* true leaves (*SI Appendix*, Fig. S5), revealing a link between cellular expansion and SCD orientation.

To directly measure the evolution of GMC shape and stomatal formation, we quantified GMC orientation, expansion, and eventual SCD orientation in 623 GMCs for 24 h from 2 dpg to 3 dpg (*SI Appendix*, Fig. S6*A*). We sorted all tracked GMCs into three groups corresponding to how well their long axes at time = 0 h (2 dpg) aligned with the average SCD angle at that position at time = 24 h (3 dpg) and then used hierarchical clustering to classify the average GMC growth and division patterns. The resulting nine clusters represented the nine possible trajectories a cell could follow from initial to final alignment (*SI Appendix*, Fig. S6 *B* and *C*). We found that the overwhelming majority (96%) of GMCs that were initially oriented along the predicted angle at that position maintained that orientation during their expansion phase and produced aligned SCDs. In contrast, half (50%) of GMCs with long axes that deviated from the cotyledon proximodistal axis by >30° reoriented through cell expansion to become more aligned by the time they underwent SCD.

### Altering Cell Growth through Cell Ablation Is Sufficient to Reorient SCD Orientation.

**Fig. 4.**
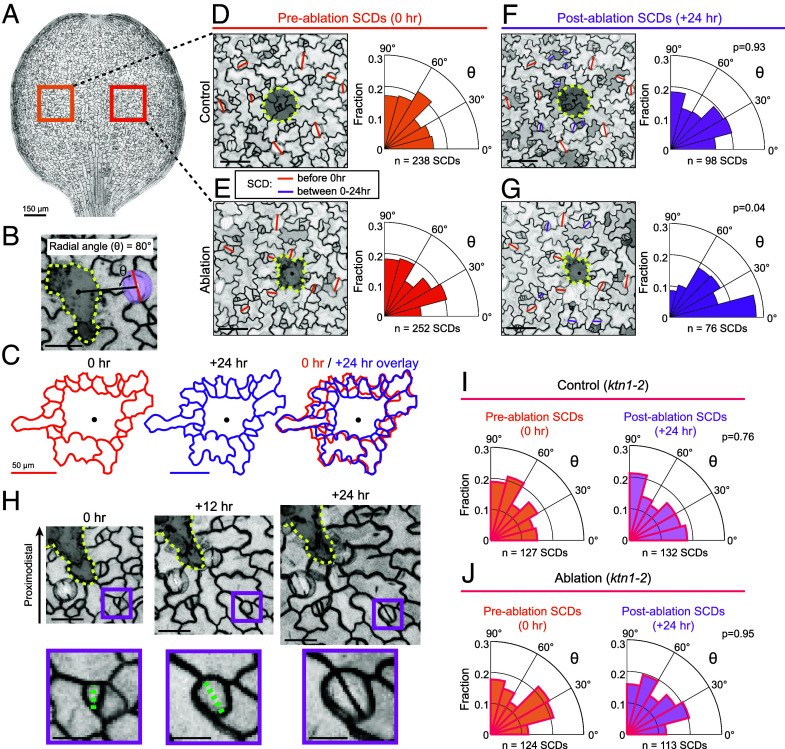
Ablations are sufficient to reorient neighboring SCDs. (*A*) 3 dpg cotyledon with the ablation and contralateral control site highlighted. (Scale bar, 150 µm.) (*B*) Illustration of how the radial angle between the ablation site (dashed yellow line) and the SCD (solid orange line) was calculated. (Scale bar, 25 µm.) (*C*) Example of the cellular front surrounding the ablation site at 0 and 24 h. (Scale bar, 150 µm.) (*D*–*G*) SCD tracking around control (*D* and *F*) and ablation sites (*E* and *G*). (*Left*) Images of the tracked areas. (*Right*) Polar histograms of the radial angles (θ). (Scale bar, 50 µm.) n = 16 cotyledons. (*H*) (*Top*) GMC reorienting its long axis toward the ablation site (dashed yellow line) and the subsequent SCD. (Scale bar, 25 µm.) (*Bottom*) Zoom of the indicated GMC and its long axis (dashed green line). (Scale bar, 10 µm.) (*I* and *J*) Polar histograms of the radial angles (θ) around control (*I*) and ablation sites (*J*) in *ktn1-2* cotyledons. n = 26 cotyledons.

Because our tracking data supported a model where GMC expansion prepatterns the oriented stomatal field, we next tested whether changing the direction of GMC growth was sufficient to reorient SCD orientation. The magnitude and direction of cellular growth are controlled not only by a cell’s internal turgor pressure and its own cell wall but also by the mechanical environment dictated by the surrounding cells (61, 62). Previous work showed that small ablations in the leaf epidermis reorient tensile stress in the neighboring cells surrounding the ablation site, triggering microtubule reorganization within hours (63). Hypothesizing that the ablation and subsequent microtubule reorganization would locally affect cell expansion, we generated small wounds on one side of the cotyledon and assayed whether this physical perturbation was sufficient to reorient SCD (Fig. 4 *A* and *B*). First, we monitored cell expansion following the ablation and noted a preferential cell expansion toward the wound edge (Fig. 4*C*). Having found that cell growth is locally reoriented by the ablation, we measured SCD alignment relative to the ablation and an identically shaped nonablated control region on the contralateral side of the same cotyledon. As expected, stomata were not aligned relative to the control or ablation site upon initial wounding (Fig. 4 *D* and *E*), and new SCDs remained similarly unaligned toward the control site after 24 h (Fig. 4*F*). In contrast, SCDs in the 24 h following the ablation were significantly reoriented toward the ablation center (Fig. 4*G*).

To analyze how the ablations caused SCD reorientation, we monitored GMC behavior in the 24 h following ablation, similar to our analysis of GMC growth and division in unperturbed 2 to 3 dpg cotyledons. We found that ablations shifted the major direction of GMC growth, reorienting GMC expansion toward the wound end (Fig. 4*H*). Finally, to confirm that this reorientation depended on changes in mechanical force, we tested whether KTN1 was required for ablation-induced SCD reorientation and found that SCDs did not reorient toward the wound in *ktn1-2* (Fig. 4 *I* and *J*). From these experiments, we concluded that SCDs are force-responsive and that altering the major direction of cell expansion is sufficient to reorient SCD.

### Genetically Induced Changes to Cellular Growth Are Sufficient to Reorient the Global SCD Pattern.

Our ablation data demonstrated that SCDs can be locally reoriented by changing the major direction of tensile stress but were not sufficient to determine whether this mechanism could generate a global pattern during leaf development. To comprehensively test this hypothesis, we created a series of *Arabidopsis* lines where the magnitude and principal direction of cell expansion could be titrated within distinct cellular populations during leaf development. First, we generated plants overexpressing *LONGIFOLIA1* (*LNG1*, also known as *TRM2*), as 35Sp::LNG1 plants were previously shown to have dramatically elongated pavement cells and leaves (64, 65). Stomatal lineage phenotypes in LNG1-overexpressing lines were not previously characterized.

**Fig. 5.**
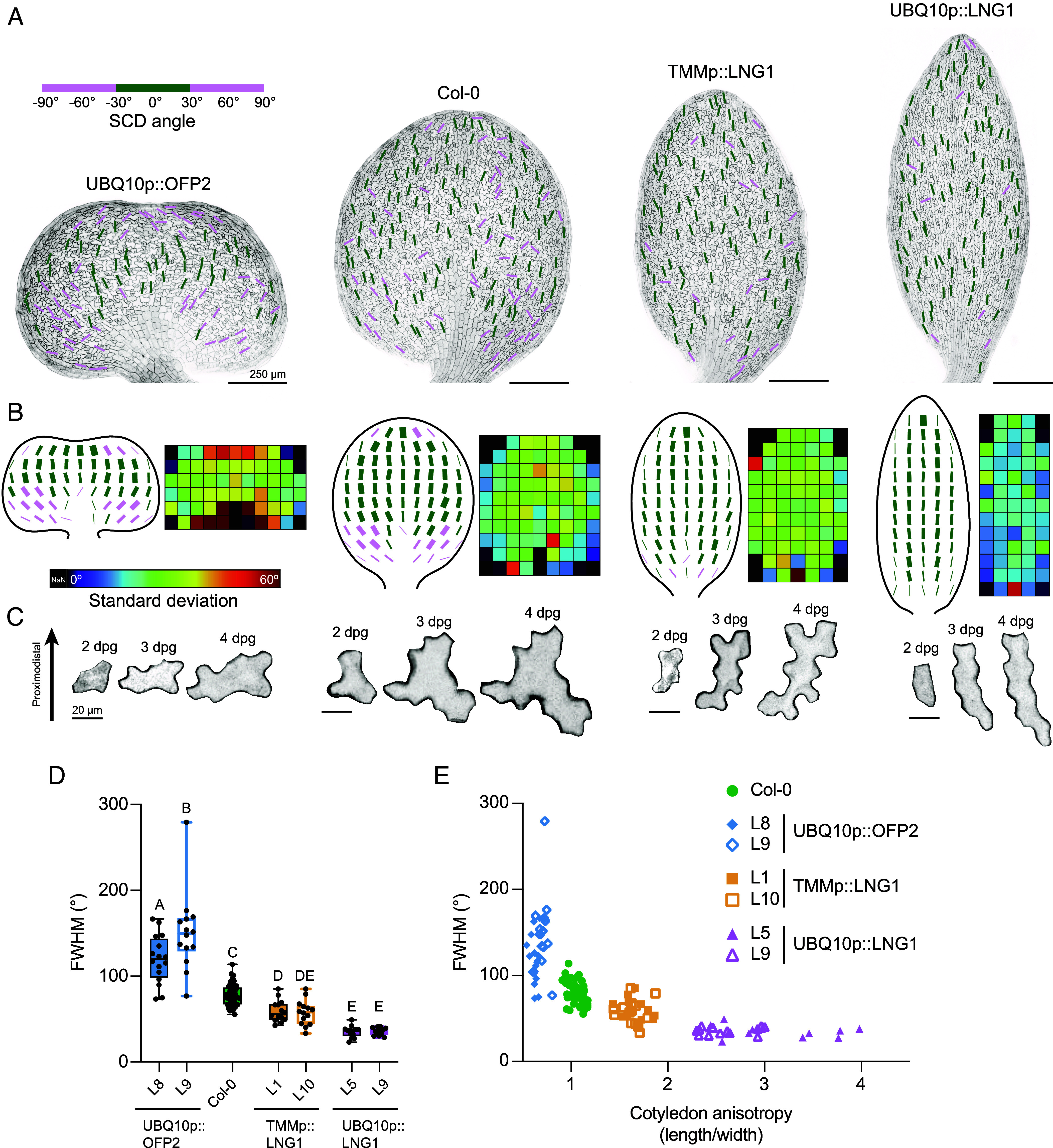
Altered expansion in neighbor cells is sufficient to reorient the polarized SCD field. (*A*) 3 dpg cotyledons from the indicated genotypes (UBQ10p::OFP2 L8, Col-0, TMMp::LNG1 L1, UBQ10p::LNG1 L9) with color-coded SCDs. (Scale bar, 250 µm.) (*B*) (*Left*) Composites of 3 dpg cotyledons shown in *A*. (*Right*) Associated maps of the corresponding angular deviations. n = 16 (UBQ10p::OFP2 L8, 52 to 138 stomata/cotyledon), 15 (Col-0, 85 to 143 stomata/cotyledon), 14 (TMMp::LNG1 L1, 72 to 200 stomata/cotyledon), and 13 (UBQ10p::LNG1 L5, 65 to 186 stomata/cotyledon) cotyledons. (*C*) Image series of typical pavement cell expansion for each genotype. (Scale bar, 20 µm.) (*D*) FWHM values from two independent 3 dpg transgenic lines for each indicated genotype. Same n values as *B* except n = 55 (Col-0, 69 to 248 stomata/cotyledon), and for additional lines, n = 14 (UBQ10p::OFP2 L9, 43 to 183 stomata/cotyledon), 14 (TMMp::LNG1 L10, 65 to 186 stomata/cotyledon), and 13 (UBQ10p::LNG1 L5, 81 to 167 stomata/cotyledon) cotyledons. (*E*) FWHM values scale with cotyledon anisotropy. Same n values as *B* and *D*.

We overexpressed *LNG1* using three different promoters: 1) the ubiquitously expressed and constitutively active *UBIQUITIN10* promoter (UBQ10p), 2) the stomatal lineage-specific *TMM* promoter (TMMp), and 3) the early stomatal lineage-specific *SPEECHLESS* (SPCHp) promoter. All three genotypes developed elongated cotyledons and true leaves but the degree of anisotropy varied based on the promoter (Fig. 5*A* and *SI Appendix*, Fig. S7*A*).

We measured SCD alignment in multiple independent transgenic lines for all three genotypes at 3 dpg and compared the FWHM values to those from Col-0 (*SI Appendix*, Fig. S7 *B* and *G*). Increasing cotyledon elongation was sufficient to reorient the SCD field, with the highly elongated UBQ10p::LNG1 cotyledons showing the highest degree of stomatal alignment (Fig. 5 and *SI Appendix*, Fig. S7*D*). This effect became more pronounced by 5 dpg as the UBQ10p::LNG1 cotyledons continued to lengthen at the expense of widening (*SI Appendix*, Fig. S7 *C* and *E*). TMMp::LNG1 cotyledons showed an intermediate phenotype, with FWHM values between those from Col-0 and UBQ10p::LNG1 (Fig. 5). Interestingly, although SPCHp::LNG1 seedlings made slightly longer cotyledons, stomatal alignment did not differ significantly from Col-0, suggesting the existence of a threshold below while increasing elongation is insufficient to alter SCD orientation (*SI Appendix*, Fig. S7 *A* and *B*).

While our ablation data demonstrated that directional cell expansion reorients SCDs, our *LNG1* overexpression lines gave us the opportunity to investigate more specifically whether SCD orientation is driven by changes in GMCs or by a nonautonomous effect of expansion in the surrounding pavement cells. We quantified GMC morphology and found that neither GMC area nor the GMC aspect ratio was changed in any of our *LNG1* lines (*SI Appendix*, Fig. S8 *A*–*C*). In stark contrast, pavement cell expansion strongly reoriented along the proximodistal axis, and the magnitude of expansion scaled with both the strength of the promoter and the degree of SCD hyperalignment (*SI Appendix*, Fig. S8 *D*, *F*, and *G*). These data indicate the global change in SCD orientation was not driven by increasing GMC anisotropy, but was due instead to a nonautonomous effect of directional cell expansion in surrounding pavement cells on the orientation of GMC long axes.

The *LNG1* overexpression lines demonstrated that hyperalignment with the proximodistal axis is tunable; the greater the anisotropic pavement cell expansion along that axis, the higher the stomatal alignment. Finally, we tested whether shifting expansion laterally could reorient the SCD field away from the proximodistal axis by overexpressing *OVATE FAMILY PROTEIN 2* (*OFP2*) under the control of the UBQ10p (UBQ10p::OFP2). These lines exhibited the phenotypes seen in previously reported *OFP* overexpression lines (66–68), with noticeably squat cotyledons and true leaves (Fig. 5*A*) and pavement cell reorientation in the lateral direction (*SI Appendix*, Fig. S8 *A*–*D* and *G*). In agreement with the results from the *LNG1* overexpression lines, changing the major direction of cotyledon expansion in UBQ10p::OFP2 was sufficient to reorient the stomatal field and was recapitulated in multiple independent transgenic lines (Fig. 5 and *SI Appendix*, Fig. S7*G*). The lateral reorientation became more pronounced over development, and the SCD distribution became bimodal by 5 dpg (*SI Appendix*, Fig. S7 *C* and *F*). Therefore, the stomatal field is plastic in *Arabidopsis* and directly tunable by altering the major axis and magnitude of pavement cell expansion.

### SCD Alignment Is Widespread across Eudicots and Is Oriented along Tissue Axes.

**Fig. 6.**
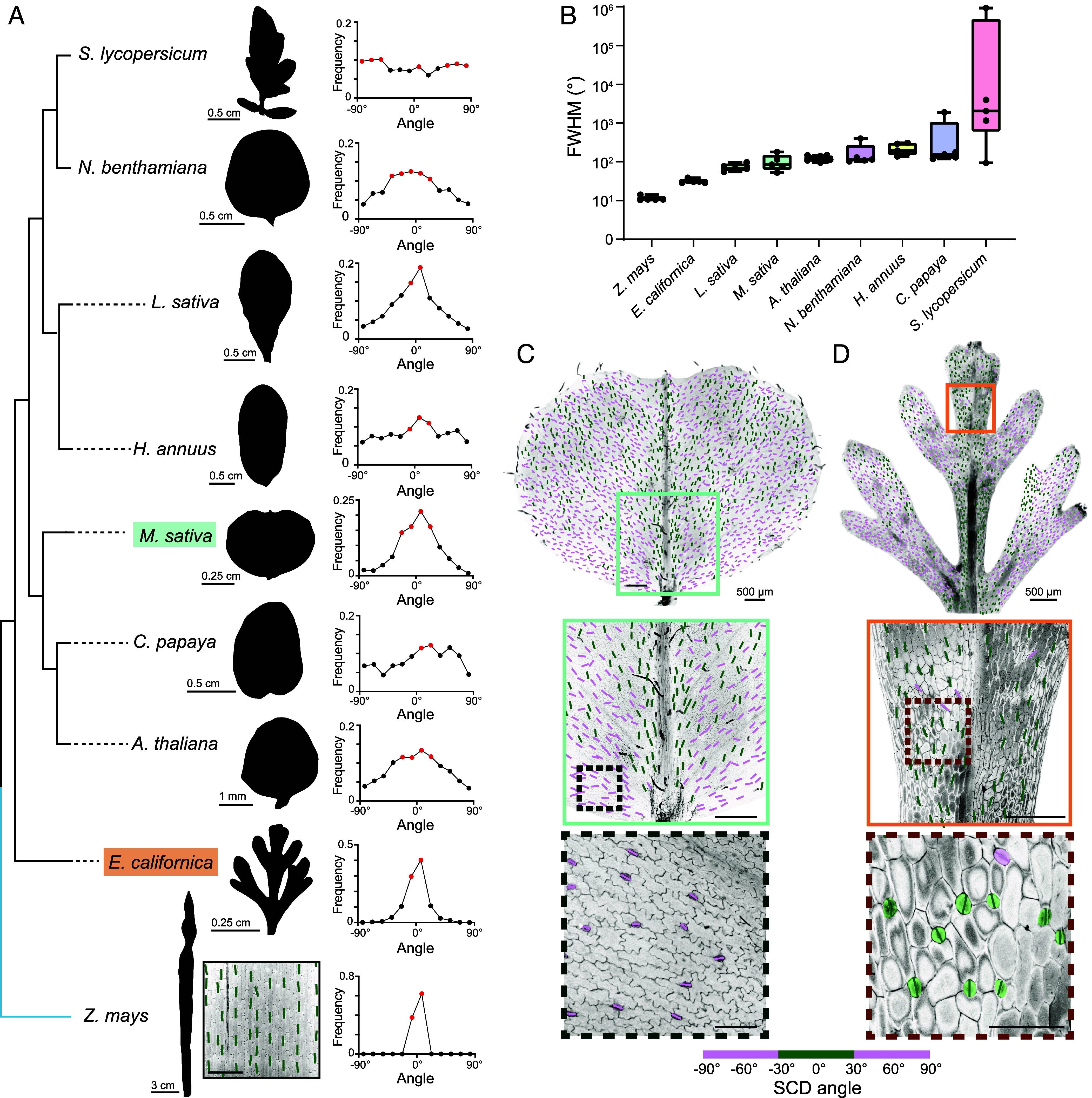
Oriented SCD fields are widespread across eudicots and align stomata to tissue axes. (*A*) (*Left*) Representative images of true leaves that were used for SCD quantification with the phylogenetic relationships between the investigated species. (*Right*) Stomatal alignment relative to the proximodistal axis along the midvein for each species. [Scale bar, 0.5 cm (*S. lycopersicum*, *N. benthamiana*, *L. sativa*, *H. annuus*, *C. papaya*), 0.25 cm (*M. sativa*, *E. californica*), 1 mm (*A. thaliana*), 3 cm (*Z. mays* whole leaf), and 300 µm (*Z. mays* zoom).] (*B*) FWHM values for indicated species. n = 5 true leaves each except *A. thaliana* (n = 11). (*C* and *D*). *M. sativa* (*C*) and *E. california* (*D*) true leaves with color-coded SCDs. [Scale bar, 500 µm (*Top* and *Middle*) and 100 µm (*Bottom*).]

The mechanism of SCD orientation we found in *Arabidopsis* could explain stomatal alignment patterns across species without invoking unknown, conserved polarity regulators. Therefore, we explored the possibility that cryptic SCD fields might be more widespread in eudicot leaves than previously appreciated by quantifying stomatal alignment in morphologically varied true leaves from seven additional eudicot species from different genera: *Carica papaya*, *Eschscholzia californica*, *Helianthus annuus*, *Lactuca sativa*, *Medicago sativa*, *Nicotiana benthamiana*, and *Solanum lycopersicum* (Fig. 6*A*). We analyzed global SCD alignment in each species, focusing on midvein-adjacent regions, and compared the measurements to the FWHM scores from both *Arabidopsis* and maize.

Polarized SCD fields were found in all of the examined species, although both the magnitude and orientation of global alignment varied significantly (Fig. 6*B*). *L. sativa*, *N. benthamiana*, *H. annuus,* and *C. papaya* displayed patterns much like *Arabidopsis*, with divisions biased along the leaf’s proximodistal axis. SCD orientation near the midvein in *S. lycopersicum* true leaves was variable, but stomata were well aligned along the direction of outgrowth in serrations, similar to what we observed in *Arabidopsis* true leaves.

Of the species we examined, we were the most intrigued by the SCD orientation fields seen in the elongated leaflets of *E. californica* and the heart-shaped leaves formed by *M. sativa* (Fig. 6 *C* and *D*) which mirrored key leaf shape features we saw in our UBQ10p::LNG1 and UBQ10p::OFP2 lines, respectively. Strikingly, stomata were hyperaligned along the proximodistal axis of each leaflet in *E. californica* while stomata in the *M. sativa* true leaf were aligned along the proximodistal axis near the midvein but fanned outward toward the leaf margins nearer to the periphery. In both species, a closer look at cell morphology revealed that stomata were aligned with the direction of pavement cell expansion. Therefore, the stomatal patterns we found in other eudicots are consistent with the force-responsive SCD orientation model we identified in *Arabidopsis*.

## Discussion

In this work, we identified a stomatal patterning mechanism that relies on orientation of the final SCD in the lineage. This regulation creates a polarized stomatal field in multiple organs, with GC orientation biased along global tissue axes. While stomatal alignment had been noted in some species for decades (23), it was generally considered to be restricted within certain genera and, importantly, the underlying mechanisms were unknown. Our finding that stomata are aligned in *Arabidopsis* leaves provided an ideal system to identify the responsible pathways.

Using time-lapse microscopy, we found that GMCs divide along their long axes, which could be explained by 1) a cellular ruler that “measures” a cell’s dimensions or 2) a mechanics-based pathway that aligns divisions along the axis of greatest strain, which in WT cells will tend toward the longest axis. We found that SCDs no longer follow the GMC long axis in *KTN1* mutants even as GMC morphology remains unchanged, supporting a force-based mode of division orientation (53). How, then, is the division plane oriented along the axis of greatest strain? Our data indicate that the microtubule network is less anisotropic and less aligned with the GMC long axis in *ktn1-2*, consistent with a role for microtubule self-organization in division orientation. One of our more puzzling findings was that GMCs continue to divide along their long axes in *trm678*, suggesting that proper preprophase band establishment is dispensable for SCD orientation. The PPB is essential for oriented stomatal lineage ACDs (51), revealing differential division requirements during stomatal development. Our finding that the PPB is not required for SCD orientation could be explained by a mechanism where interphase arrays influence division orientation independent of PPB establishment, which is supported by some recent work (69). Alternatively, ultrastructure studies have found that the cell walls are slightly thicker at the poles along the GMC long axis (70). Whether microtubule alignment along the long axis is responsible for this cell wall thickening and whether cell wall anisotropy is immediately upstream of division orientation will need to be tested in future studies.

By tracking cell and leaf expansion over time, we showed that GMC growth along the principal direction of leaf expansion primes the SCD field. Accordingly, mutants that alter leaf shape reorient SCD orientation. A key, outstanding question remains how the growth of individual cells is coordinated with growth of the developing organ, a problem where computational modeling could prove useful. Notably, while we were preparing our results for publication, another group used largely complementary methods to independently identify the global SCD orientation field in *Arabidopsis* cotyledons (71). They noted a difference in stomata alignment on the abaxial and adaxial surfaces, which we also described in our work, and used these differences to develop a mechanics-based model for SCD orientation that largely concurs with our own findings.

Proper division orientation is one of the pillars of morphogenesis. In some cases, such as in the dome-shaped shoot apical meristem, oriented divisions control overall organ shape (53). The oriented SCDs we described here are notably different in this regard. We have not found any evidence to suggest that stomatal lineage SCDs impact overall organ (e.g., cotyledon, true leaf) morphology. Instead, when SCDs are misoriented, as they are in *ktn1-2*, they create aberrantly shaped stomata with small pores. Interestingly, malformed stomata were observed in a previous study (72), and our data identify the underlying defect. Why misoriented SCDs lead to GC morphology and pore defects remains to be determined. Finally, how the increased randomization of SCD orientation in *ktn1-2* affects stomatal kinetics remains to be seen. It is tempting to speculate that global stomatal alignment could influence overall stomatal physiology by impacting pore opening and closing efficiency. By identifying additional genes required for SCD orientation in the leaf epidermis, we can begin to test how the polarized stomatal field influences gas exchange to regulate photosynthesis and plant growth.

## Materials and Methods

### Plant Material and Growth Conditions.

All *A. thaliana* lines generated and used in this study were in the Col-0 background. A complete description of the lines used in the study can be found in *SI Appendix, Materials and Methods*. *Arabidopsis* seeds were sterilized in 20% bleach for 10 min, followed by 3 rinses with Milli-Q water. Seeds were then sown on 1/2 MS + 0.5% sucrose + 0.7% agar plates and stratified in the dark at 4 °C for at least 2 d. The day a plate was moved into the growth chamber was counted as 0 dpg. The growth chamber (Percival, Model CU41L5) was set to long-day conditions (16 h light, 8 h dark) at 22 °C with SciWhite^TM^ LED lighting at 650 lx. The IAA experiments and Col-0 needle ablations were performed in the same Percival with identical light conditions but set to 25 °C.

### Generation of Plasmids for Plant Transformation.

TMMp::LNG1, SPCHp::LNG1, UBQ10p::LNG1, and UBQ10p::OFP2 were all generated using Gateway cloning (Invitrogen). LNG1 (AT5G15580) was amplified from *Arabidopsis* genomic DNA using CACCCAACAACCTTCTGAGGCCAGAG and GGGGTTCAGAGAACCAAGAAACC and ligated into pENTR D-TOPO. OFP2 (AT2G30400) was amplified from *Arabidopsis* genomic DNA using CACCATGGGGAATTACAAGTTCAG and TTACTTTGTTTTTGTAAGTTG and ligated into pENTR D-TOPO. The TMM and SPCH promoter sequences were in pDONR P4-P1R (73) and the UBQ10 promoter sequence was in pENTR 5′ TOPO. Vectors were assembled in R4pGWB601 (74). All constructs were validated by Sanger sequencing.

### Transgenic Line Generation.

To generate LNG1- and OFP2-overexpressing lines, ML1p::mCherry-RCI2A plants were transformed using the agrobacterium-mediated floral dip method (75) in strain GV3101. Additional information about line selection can be found in *SI Appendix*, *Materials and Methods*.

### Confocal Microscopy.

All confocal microscopy, with the exception of the images of microtubule organization in GMCs and GCs, was done on a Leica Stellaris 5 with HyD detectors using the 10×/0.40NA or 20×/0.75NA objectives. Images of microtubules were obtained on a Nikon Eclipse Ti2-E microscope with a CSU-X1 spinning disk, a Prime 95B sCMOS camera and the Plan Apo ʎ 60× NA 1.40 oil objective. Additional details can be found in *SI Appendix*, *Materials and Methods*.

### Image Analysis.

Analyses were performed using Fiji and Matlab. For details, see *SI Appendix,* *Materials and Methods*.

### SCD Visualization, Simulation, and Averaging.

The image overlays with SCDs color-coded by angle were generated in MATLAB using annotation coordinates obtained in Fiji. For visualization purposes, the length of each SCD line was set to a user-defined length. A detailed description of the methods used to simulate random cotyledons and generate the averaged cotyledon images can be found in *SI Appendix*, *Materials and Methods*.

### FWHM Global Alignment Analysis.

FWHM was calculated in MATLAB. A one-term Gaussian model was fit to the histogram of SCD angles, yielding the following model: f(x) = a * e^−[(x-b)/c]^2^. FWHM was then calculated using the following equation: FWHM = 2 * sqrt [log(2)] * c.

### Hormone Treatments.

For the IAA experiments, seeds were sown on 1/2 MS media with 0.5% sucrose containing either 500 nM or 1 µM IAA (Thermo Scientific Chemicals, AC122160100) or EtOH and grown at 25 °C. For the NPA experiments, seeds were sown on 1/2 MS media with 0.5% sucrose containing either 10 µM NPA (Tokyo Chemical Industry, N0067) or DMSO and grown at 22 °C.

### Hierarchical Cluster Analysis.

Cotyledons of intact 2 dpg seedlings were imaged at 12 h intervals, for a total of three images per sample. A detailed description of the hierarchical cluster analysis can be found in *SI Appendix*, *Materials and Methods*.

### Quantification and Statistical Analysis.

Gene sequences were obtained from The Arabidopsis Information Resource (TAIR). Statistical analysis and graph creation was done using GraphPad Prism (version 10.2.3). The means of two groups were compared using an unpaired *t* test. For comparing multiple groups, one-way ANOVA was used. If there was a significant difference between any of the means (*P*-value < 0.05), a Dunnett test was performed to compare the mean of each group with the control group’s mean (Col-0). The distributions of SCDs at 0 h and 24 h in the ablation experiments were compared using Kolmogorov–Smirnov tests. Error bars represent the SD. For all graphs: ns—not significant, *P < 0.05, **P < 0.01, ***P < 0.001. Hierarchical clustering was performed in R and Rstudio (version 2023.06.1 + 524.pro1) using the tidyverse and gghighlight packages. Image analysis was performed with Fiji/ImageJ (version 2.14.0/1.54f). Analyses done in MATLAB utilized release R2024a.

## Supplementary Material

Appendix 01 (PDF)

## Data Availability

All study data are included in the article and/or *SI Appendix*.
