# Supplementary material for: Force-responsive symmetric cell divisions orient stomata along global tissue axes
Source: Proc Natl Acad Sci U S A. 2026 Jun 30;123(27):e2529768123. doi: 10.1073/pnas.2529768123 (PMC13342852; doi:10.1073/pnas.2529768123)
Supplement: Supplementary file 1 — Appendix 01 (PDF) [file pnas.2529768123.sapp.pdf]

PNAS

Supporting Information for
Force-responsive symmetric cell division orient stomata along global tissue axes

Kensington S. Hartman, Bianca Y. Lopez, Juan H. Gonzalez, Madison E. Goetz, Aviel Cleveland, Andrew Muroyama

Andrew Muroyama
Email: amuroyama@ucsd.edu

This PDF file includes:

Supporting text – Materials and Methods
Figures S1 to S8
SI References

Supplementary Information Materials and Methods

***Arabidopsis* lines**

ML1p::mCherry-RCI2A (1), BRXL2p::BRXL2-YFP ML1p::mCherry-RCI2A (2), BASLp::YFP-BASL 35Sp::PIP2A-RFP (2), BRXL2p::BRXL2-YFP TMMp::mCherry-TUA5 (3), *basl-2* (WiscDsLox264F02) (4) 35Sp::PIP2A-RFP, *brx-q (2)*, *tmm-1 (5)*, *flp-1 myb88 (6)*, *trm678 (7)*, *ktn1-2* (SAIL_343_D12) (8) were published previously. *kan1-11 kan2-5 (9)* (AT5G16560 and AT1G32240, CS67888), *rot3-1 (10)* (AT4G36380, CS3727), *dot3-2 (11)* (AT5G10250, SALK_107193), *dot5-2 (11)* (AT1G13290, SALK_148869), *scf-9 (12)* (AT5G13300, SALK_069166), *ktn1-20 (13)* (CS73507) and *lue1 (14)* (CS9531) were acquired from the Arabidopsis Biological Resource Center at Ohio State University and the ML1p::mCherry-RIC2A reporter was introduced by crossing as indicated. *opl1-1 opl2-3 opl3-1 opl4-1 (15)* (*opl-quad*) and *fama-1 (16)* were previously published and kindly provided by Dr. Dominique Bergmann (Stanford/HHMI). *tir1-1 afb2-3 afb3-4* was published previously (17) and kindly provided by Dr. Mark Estelle (UCSD).

ktn1-2, *kan1-11 kan2-5* and *rot3-1* were confirmed by PCR. *ktn1-2* was genotyped by using PCR followed by gel electrophoresis using the following primers: 5'-TAGCATCTGAATTTCATAACCAATCTCGATACAC-3' (LB3), CACACAAGAAAAGGCACACATC (LP) and CCTTCAGGGTATTCGAAGACC (RP). *kan1-11* was genotyped by first amplifying the mutation-harboring locus via PCR using CTCTCCAGTTTGTCATCTG and ACCACTCAACTTTAGGGTTC and sequencing using CATCTGTAATTCTGTATC for a missense mutation in the 3rd exon (R272Q). *kan2-5* was amplified with GGTTCATCATCTGTGGAAACCG and CGAGTAATTCAACGGCGTGAAC and sent for Sanger sequencing with GTAAACCATCATCGACATGG to check for the presence of the nonsense mutation (C268A). *rot3-1* was genotyped using PCR followed by gel electrophoresis with the following primers: CGAGACAAAACGGCCTAAGC and AAGTTTAGGGTTTCTCCGATCACC. *scf-9* and *tir1-1 afb2-3 afb3-4* were confirmed by mounting analyzed seedlings in Hoyer's solution and confirming the previously described vein morphology defects.

Transgenic line generation

Transformants were selected on ½ MS plates with 150 μM PPT and no sucrose, and lines with single integrations were identified in the T2 generation by quantifying seedling survival on PPT-containing plates. The following are the associated counts for each LNG1/OFP2 line: SPCHp::LNG1—21 transformants, 11 lines; TMMp::LNG1—16 transformants, 9 lines; UBQ10p::LNG1—27

transformants, 8 lines; UBQ10p::OFP2—13 transformants, 9 lines. For the imaging assays, all samples came from T2 and T3 generations.

Non-*Arabidopsis* species

Tobacco (*N. benthamiana*), tomato (*S. lycopersicum* cv. M82) and maize (*Z. mays*, B73) were generously provided by Dr. Alexandra Jazz Dickinson (UCSD). Alfalfa (*M. sativa*, accession W6 2502) was a kind gift from Dr. Alisa Huffaker (UCSD). Lettuce (*L. sativa* cv. Parris Island cos), California poppy (*E. californica* cv. aurantiaca orange), and sunflower (*H. annuus* cv. Russian mammoth) were purchased from Home Depot (San Diego, CA). Papaya (*C. papaya*, 'Mexican') seeds were harvested from a papaya fruit bought at a local grocery store.

Confocal microscopy

For *Arabidopsis* lines without genetically encoded plasma membrane markers, cell walls were stained by incubating samples in propidium iodide (PI, 10 μg/ml) for 5 min. For non-*Arabidopsis* species, cell walls were stained by incubating in PI (10-200 μg/ml, higher concentration used for larger samples) for 30 min-7 hr or with FM4-64 (20-40 μM) for 2-7 h (alfalfa, lettuce, maize, sunflower, tomato). PI and YFP reporters were excited by a 514 nm laser and their emissions were detected between 520-650 nm. FM4-64 and mCherry reporters were excited by a 561 nm laser and their emissions were detected between 565-650 nm.

Time-lapse data were acquired with 90 min intervals and a duration of 13-15 h using the 20x objective at 2x zoom. Whole seedlings were mounted on a coverslip and covered with a 0.15 mm HybriWell chamber (Grace Bio-Labs, HBW75) connected to a pump (SyringePump.com, NE-300 Just Infusion™ Syringe Pump) flowing ½ MS liquid media at 0.04 ml/min. For manual time course imaging (images acquired 12 or 24 h apart), whole seedlings were gently placed on and removed from slides for imaging and were placed back on plates in the growth chamber between imaging sessions. Whole leaf images (Figure 6) were acquired on a Samsung Galaxy phone camera. Sepal mounting was performed per the protocol published in He et al (18).

Image analysis

SCD orientation relative to the leaf's proximodistal axis was measured by manually annotating the cell wall separating paired guard cells with a pore using the "Straight Line" tool. GMCs were measured by manually tracing the cell outline

with the “Polygon tool” and fitting an ellipse to find the long axis, aspect ratio, and area. When comparing GMC morphology with corresponding division angle, the GMC was measured in the time frame immediately preceding the time frame in which the division first appears (i.e., 90 min before for Col-0 and *ktn1-2* movies, 60 min for *trm678* movies). The difference between the GMC long axis and SCD angle was always calculated as the acute angle; to accommodate this, the range of the angles was extended up to 120°.

To measure whole cotyledon anisotropy, the outline of each sample (with the petiole trimmed from the image) was first extracted using Fiji’s “Analyze Particles” tool. For most samples, anisotropy was defined as the AR calculated by fitting an ellipse to this outline. For samples that were visually wider than they were long, anisotropy was defined as the inverse of the AR. Finally, for samples where the calculated longest axis of the fit ellipse did not align with either the proximodistal or lateral axis, the length of the sample along these two axes was manually measured using Fiji’s “Straight Line” tool, and the anisotropy was defined as the proximodistal length divided by the lateral length.

Quantification of microtubule anisotropy and major axis was performed using the FibrilTool plugin for Fiji (19). Pore size was analyzed by manually tracing the pore aperture using the “Polygon” tool in Fiji from maximum projections of the ML1p::mCherry-RCI2A reporter.

SCD simulation and averaging

Simulations of cotyledons with randomly oriented SCDs were generated in MATLAB. For each of 25 generated samples, an ellipse of random length and width was generated from the normal distribution of 3 dpg cotyledon lengths and aspect ratios using normrnd(), yielding the simulated cotyledon outline. 150 (the average stomatal number at 3 dpg) random SCD angles were generated from a uniform distribution ranging from -90° to 90° using rand(). The x- and y-coordinates at one end of each SCD were generated from a uniform distribution spanning the width or length of the sample’s outline using random(‘Uniform’) and the coordinates at the other end were computed from those coordinates plus cosine or sine of the paired, randomly generated SCD angle. If an SCD’s coordinates fell outside the sample’s elliptical outline, new random coordinates were drawn.

MATLAB was also used to find the average SCD angles by position on the leaf, which were used to create composite cotyledons. For each sample, a 9x10 grid was fitted to the cotyledon’s outline. For each grid space, the angles of SCDs inside its bounds were stored with those of the same grid space on all the other samples of the same genotype. SCDs straddling two grid spaces were counted in both spaces. Because this analysis is concerned with orientation but not direction (i.e., opposite directions are the same orientation), the aggregated angles were

converted to unimodal orientation (e.g., 90° is now considered the same as -90°) using circ_axial(p = 2) from the Circular Statistics Toolbox (20). The circular averages were then calculated using circ_mean(), yielding the average orientation of all samples within each grid space. The angular deviation of angles within each grid space was also calculated using circ_std(). For plotting the averages, the thickness of each line was weighted by the number of SCDs that fell within that grid space, and for the 3 dpg and simulated random composites in Figure 1, the length of each line was scaled by the mean resultant vector length (calculated using circ_r()). In Figure 1: For the real composite, each grid space averaged the orientation of 1-114 SCDs (with a mean of 47 SCDs and standard deviation of 24 SCDs); and for the simulated randomized composite, each grid space averaged the orientation of 1-70 SCDs (with a mean of 46 SCDs and standard deviation of 16 SCDs). In Figure 5: For the UBQ10p::OFP2 composite, each grid space averaged the orientation of 1-57 SCDs (with a mean of 27 SCDs and standard deviation of 15 SCDs); for the Col-0 composite, each grid space averaged the orientation of 1-47 SCDs (with a mean of 26 SCDs and standard deviation of 12 SCDs); for the TMMp::LNG1 composite, each grid space averaged the orientation of 1-67 SCDs (with a mean of 28 SCDs and standard deviation of 13 SCDs); and for the UBQ10p::LNG1 composite, each grid space averaged the orientation of 0-62 SCDs (with a mean of 24 SCDs and standard deviation of 14 SCDs).

AFT local alignment analysis

Alignment by Fourier Transform (AFT) was performed using the Matlab package described in Marcotti et al (21). The input images were generated in Fiji by flattening the manually annotated ROIs of the shared guard cell walls onto a blank image of the same dimensions as the original confocal image. When running 'AFT_batch,' the 'Neighborhood size' was set to the average area that contained an average of three stomata for a given genotype, developmental stage, or species. The 'Window overlap' was kept at the default 50% and the 'Neighborhood radius' was adjusted as needed. For pairwise comparisons in this manuscript, 'Neighborhood radius' was set to 3.

Hierarchical cluster analysis

GMC long axis and SCD orientation relative to the proximodistal axis were first measured in Fiji. Then, for each GMC and SCD, the orientation relative to the average SCD angle at its position on the leaf at 3 dpg was calculated in MATLAB: first, each GMC and SCD was assigned to a grid space (i.e., position on the leaf), using the same method as was used for assigning SCDs when calculating the

average, composite cotyledons (see Image Analysis); next, the difference between the measured GMC/SCD angle and the average 3 dpg SCD angle at the corresponding leaf position was calculated, yielding the GMC's/SCD's orientation relative to that average. To perform hierarchical clustering, cells were first classified into three groups based on the alignment of the GMC long axis at time = 0 hrs (2 dpg) with the average 3 dpg SCD angle at that position: aligned (0-30 degrees), mid-aligned (30-60 degrees), and unaligned (60-90 degrees). For each group, the Euclidean distance metric of each cell's angle over time was calculated using the dist() function in R. The hclust() function was then run on the Euclidean distance metric for each group, using the UPGMA agglomeration method. Hierarchical clustering groups together similar trajectories without inherently defining the number of clusters, requiring the user to define the number of clusters after the fact. At 24 h, SCDs will fall within one of the three ranges defined initially as aligned, mid-aligned, or unaligned. Based on the prediction that each group might naturally cluster into those three ranges for the 24 h SCD angle, the number of clusters was set to three. GMCs that started in the 0-30° cluster were considered to have maintained their orientation if they ended in the same SCD cluster (0-30°). Initially misoriented GMCs were considered to have reoriented toward the proximodistal axis if they started in the 30-60° cluster and ended in the 0-30° cluster or if they started in the 60-90° cluster and ended in either the 0-30° or 30-60° clusters.

Needle ablation

Small regions of cells, between 2724 μm^2 and 19,306 μm^2, on the abaxial epidermis of 3 dpg cotyledons were ablated using a 0.25 mm tungsten needle (Roboz Surgical Instruments, RS-6064). Identically sized, non-damaged regions on the contralateral side of the same leaf were used as controls. Immediately after ablation, seedlings were mounted on slides and cotyledons were imaged to capture the orientation of existing GMCs and SCDs at 0 h. After 24 h in the growth chamber, cotyledons were imaged again, to capture the orientation of SCDs occurring within the previous 24 hours.

The orientation of GMCs and SCDs were measured relative to the cotyledon's proximodistal axis using Fiji. A MATLAB script was then used to generate the "radial angle," the angle between the SCD's angle relative to the leaf axis and the angle of a line from the center of the SCD to the ablation or control center. For 0 h, the ablation/control center was determined with MATLAB by fitting an ellipse to the region's outline. For 24 h, the center was manually measured in Fiji by tracking cell landmarks between 0 and 24 h.

To visualize cell growth around the ablation site, the layer of cells immediately surrounding the ablation at 0 h and 24 h was traced in Illustrator. To account for overall expansion of the leaf, the 24 h trace was scaled down to fit within the same size bounding circle that fit around the 0 h trace, and the traces were overlaid by aligning the coordinates of the ablation center at both time points.

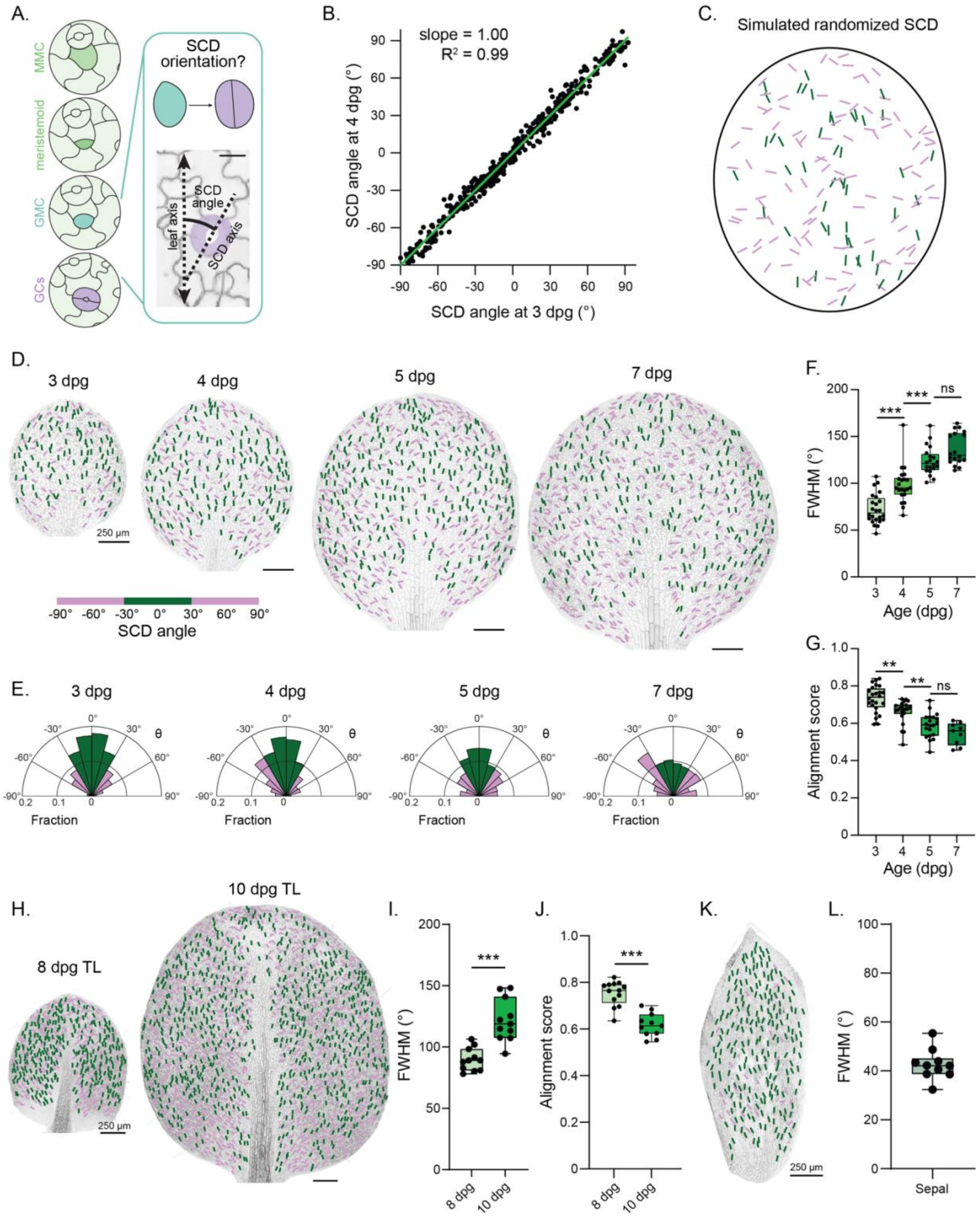

Fig. S1: The polarized SCD field is developmentally regulated and is found across other stomata-containing tissues.

A. (Left) Schematic of the *Arabidopsis* stomatal lineage. (Right) Method to measure stomatal alignment to the proximodistal axis. Scale bar – 20μm.
B. Comparison of SCD angles of paired stomata tracked from 3 dpg to 4 dpg. n = 490 cells.
C. Example of a simulated cotyledon with randomized, color-coded SCDs.
D. Representative images of ML1p::mCherry-RCI2A-expressing cotyledons with color-coded SCDs at the indicated developmental stages. Scale bars – 250 μm.
E. Polar histograms of SCD orientation relative to the proximodistal axis (0°) for the examples in (D).
F. FWHM values across cotyledon development. n = 24 (3 dpg, 81-221 stomata per cotyledon), 22 (4 dpg, 173-435 stomata per cotyledon), 20 (5 dpg, 255-587 stomata per cotyledon), 18 (7 dpg, 391-737 stomata per cotyledon) cotyledons. Note that the 3 dpg data are the same as those in Figure 1E.
G. Alignment scores across cotyledon development. Same n values as (E) except for 7 dpg (n = 9).
H. Representative images of 8 and 10 dpg true leaves (TLs) with color-coded SCDs. Scale bars – 250 μm.
I. FWHM values for 8 and 10 dpg true leaves. Note that the 10 dpg data is the same as those shown in Figure 7B. n = 11 true leaves each.
J. Alignment scores for the 8 and 10 dpg true leaves in (H). n = 13 (8 dpg) and 11 (10 dpg) cotyledons.
K. Representative image of a sepal with color-coded SCDs. Scale bar – 250 μm.
L. FWHM values for sepals. n = 10.

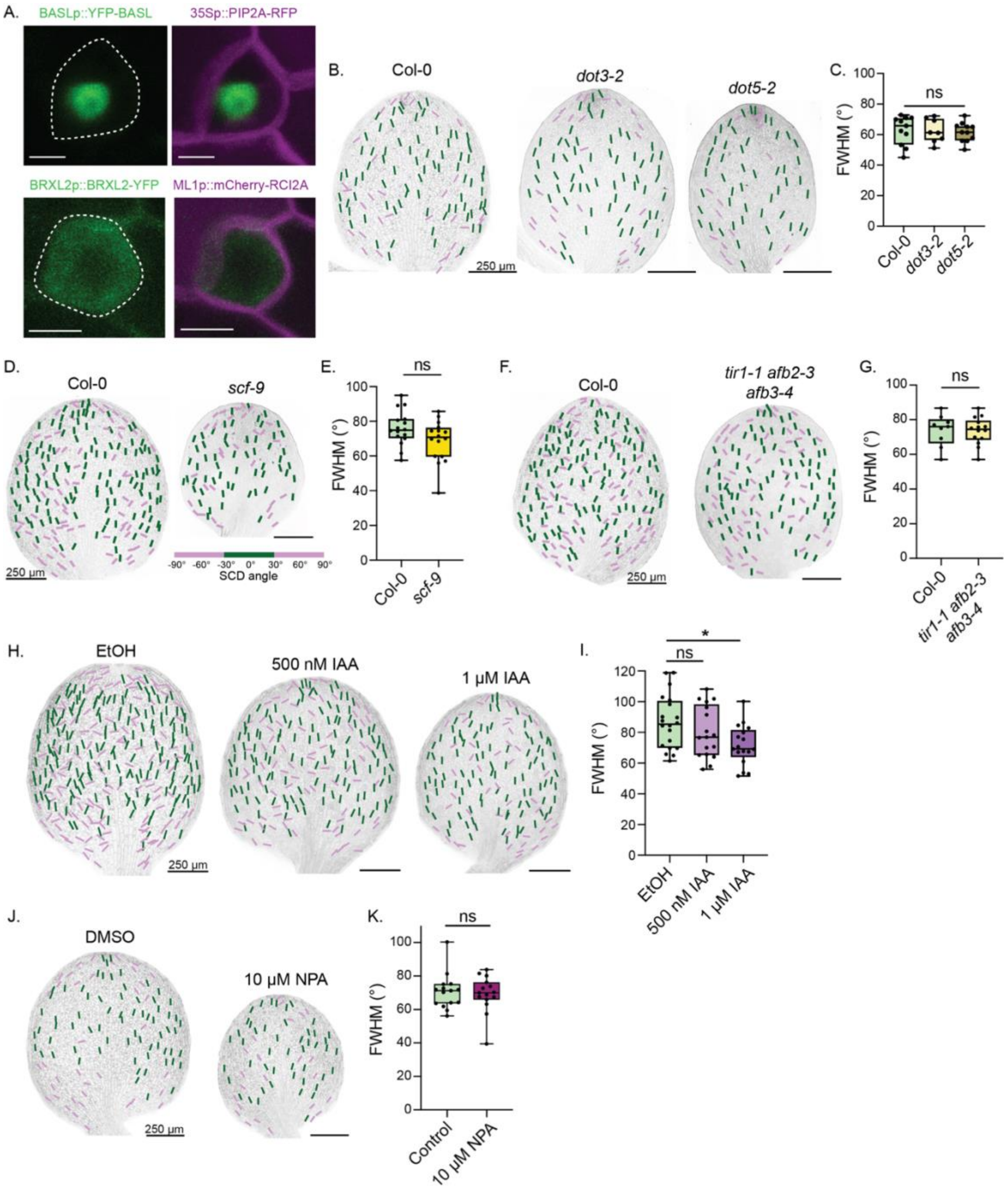

Fig. S2: ACD polar proteins, vein patterning, and auxin do not control the orientation of the SCD field.

A. Representative images of the BASLp::YFP-BASL (top) and BRXL2p::BRXL2-YFP (bottom) reporters in GMCs. Scale bars – 5 μm.
B. Representative images of 3 dpg Col-0, *dot3*-2 and *dot5-2* cotyledons with color-coded SCDs. Scale bars – 250 μm.
C. FWHM values for Col-0, *dot3-2* and *dot5-2*. n = 11 (Col-0), 8 (*dot3*-2) and 11 (*dot5*-2) cotyledons.

D. Representative images of 3 dpg Col-0 and *scf-9* cotyledons with color-coded SCDs. Scale bars – 250 μm.
E. FWHM values for Col-0 and *scf-9*. n = 16 cotyledons each.
F. Representative images of 3 dpg Col-0 and *tir1-1 afb2-3 afb3-4* cotyledons with color-coded SCDs. Scale bars – 250 μm.
G. FWHM values for Col-0 and *tir1-1 afb2-3 afb3-4*. n = 9 (Col-0) and 14 (*tir1-1 afb2-3 afb3-4*) cotyledons.
H. Representative images of 3 dpg cotyledons from seedlings grown on plates with the indicated pharmacological treatments (EtOH as control, 500 nM IAA, and 1 mM IAA). Scale bars – 250 μm.
I. FWHM values for 3 dpg cotyledons from seedlings grown on plates with the indicated pharmacological treatments. n = 20 (EtOH) and 17 (500 nM IAA and 1 mM IAA) cotyledons.
J. Representative images of 3 dpg cotyledons from seedlings grown on plates with the indicated pharmacological treatments (DMSO as control and 10 mM NPA). Scale bars – 250 μm.
K. FWHM values for 3 dpg cotyledons from seedlings grown on plates with the indicated pharmacological treatments. n = 15 cotyledons each.

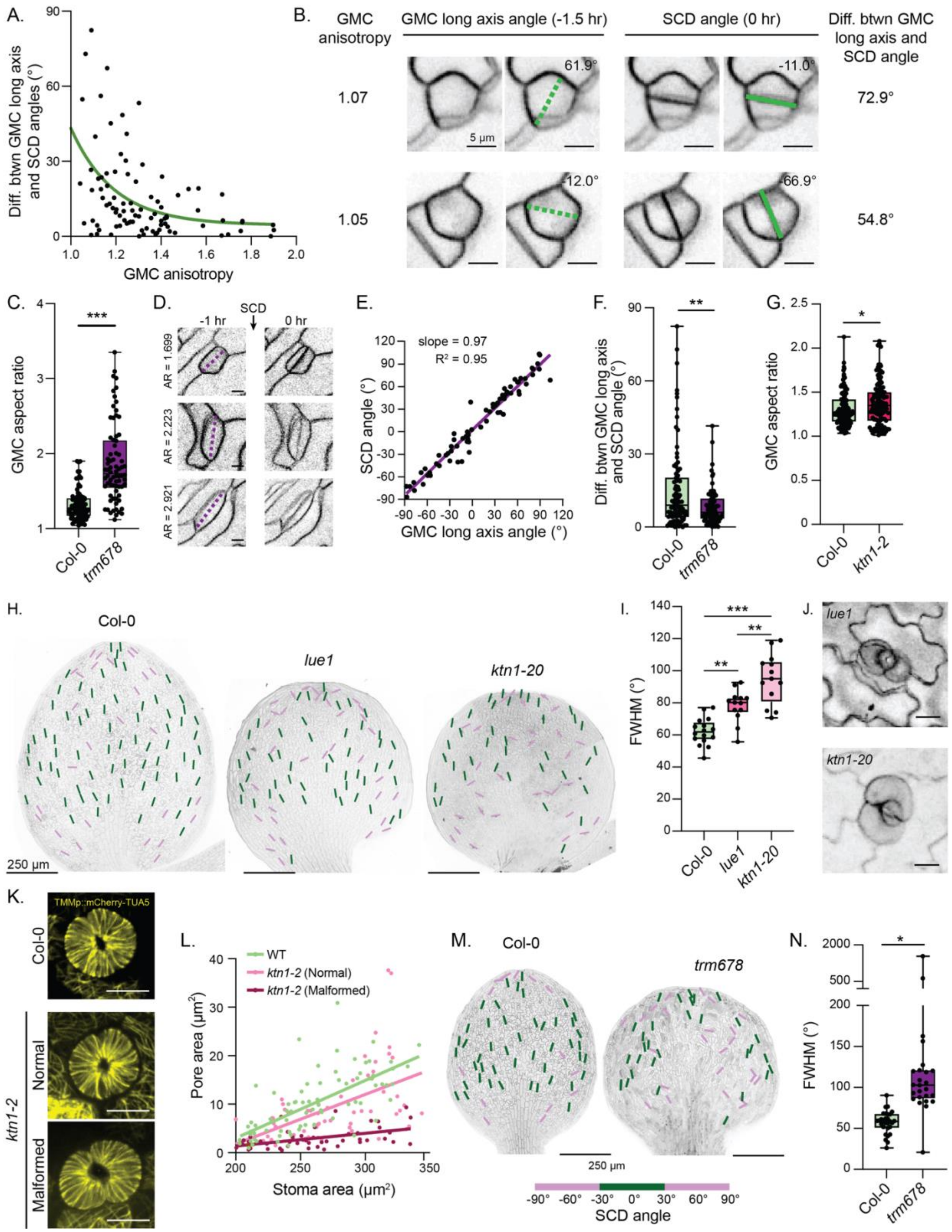

Fig. S3: Additional characterization of the relationship between GMC morphology, SCD orientation, and stomatal pore formation.

A. The difference between the GMC long axis angle and SCD angle as a function of GMC aspect ratio (green line – non-linear fit (one-phase decay)). n = 91 cells.
B. Representative images of nearly isotropic GMCs where the SCD (right, solid green line) deviates significantly from the GMC long axis (left, dashed green line). Scale bars – 5 μm.
C. Quantification of GMC aspect ratio in Col-0 and *trm678* GMCs. n = 91 (Col-0) and 74 (*trm678*) cells.
D. Representative images of SCDs in *trm678* GMCs. The dashed purple line indicates the GMC long axis immediately before mitosis. Scale bars – 5 μm.
E. Comparison of the angle of the GMC long axis immediately before mitosis to the associated SCD angle in *trm678* (purple line – linear fit). n = 74 cells.
F. Differences between the GMC long axis angle and SCD angle in Col-0 and *trm678*. Note that the data for Col-0 are the same as those in Figure 3H. n = 91 (Col-0) and 74 (*trm678*) cells.
G. Quantification of GMC aspect ratio in Col-0 and *ktn1-2* GMCs. n = 125 (Col-0) and 124 (*ktn1-2*) GMCs.
H. Representative images of 3 dpg Col-0, *lue1* and *ktn1-20* cotyledons with color-coded SCDs. Scale bars – 250 μm.
I. FWHM values for Col-0, *lue1* and *ktn1-20*. n =16 (Col-0), 13 (*lue1*) and 13 (*ktn1-20*) cotyledons.
J. Example images of malformed stoma in *lue1* (top) and *ktn1-20* (bottom). Scale bars – 10 μm.
K. Microtubule organization (TMMp::mCherry-TUA5) in Col-0 and normal and malformed *ktn1-2* stomata. Scale bars – 10 μm.
L. Comparison of the stoma and pore areas in Col-0 and *ktn1-2*. n = 76 (Col-0), 57 (normal, *ktn1-2*), and 50 (malformed, *ktn1-2*) pores.
M. Representative images of 3 dpg Col-0 and *trm678* cotyledons with color-coded SCDs. Scale bars – 250 μm.
N. FWHM values for Col-0 and *trm678*. n = 29 (Col-0) and 25 (*trm678*) cotyledons.

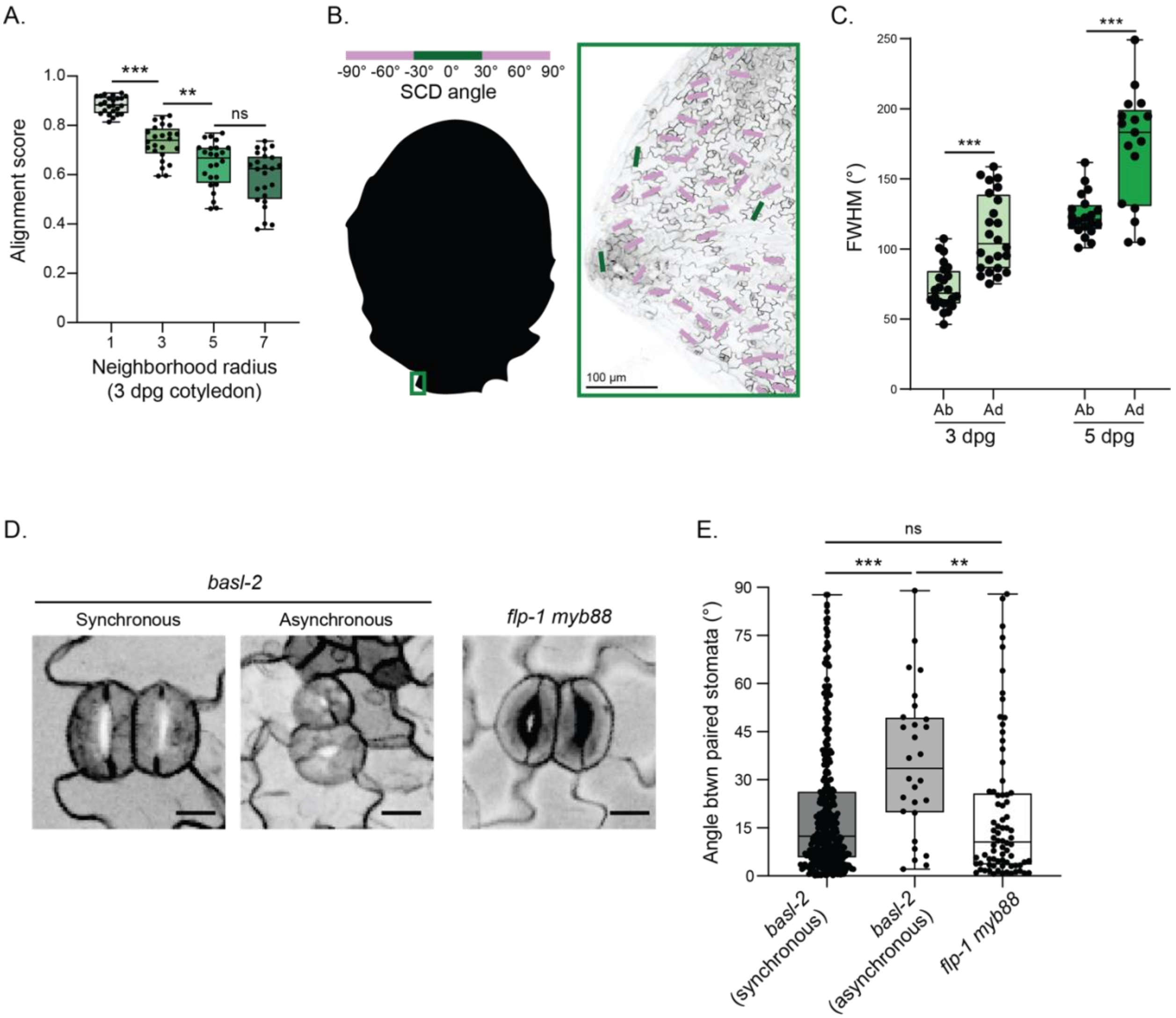

Fig. S4: Additional characterization of Col-0 and stomatal cluster mutants implicates cell expansion as an important driver of SCD orientation.

A. Alignment scores by varying neighborhood sizes in 3 dpg cotyledons. n = 24 cotyledons.
B. Example image of an SCD field oriented toward the tip of a developing serration of an *Arabidopsis* true leaf. Scale bar – 100 μm.
C. FHWM values for abaxial and adaxial SCDs at both 3 dpg and 5 dpg. n = 24 (3 dpg Ab), 24 (3 dpg Ad), 20 (5 dpg Ab) and 17 (5 dpg Ad) cotyledons. Note that the 3 dpg abaxial data are the same as those shown in Figure 1E, and the 5 dpg abaxial data are the same as those shown in Figure S1E.
D. Representative images of stomatal pairs in *basl-2* and *flp myb88*. Scale bars – 10 μm.
E. Differences between the orientation of paired stomata in *basl-2* and *flp myb88*. n = 352 (synchronous, *basl-2*), 28 (asynchronous, *basl-2*) and 76 (*flp myb88*) stomatal pairs.

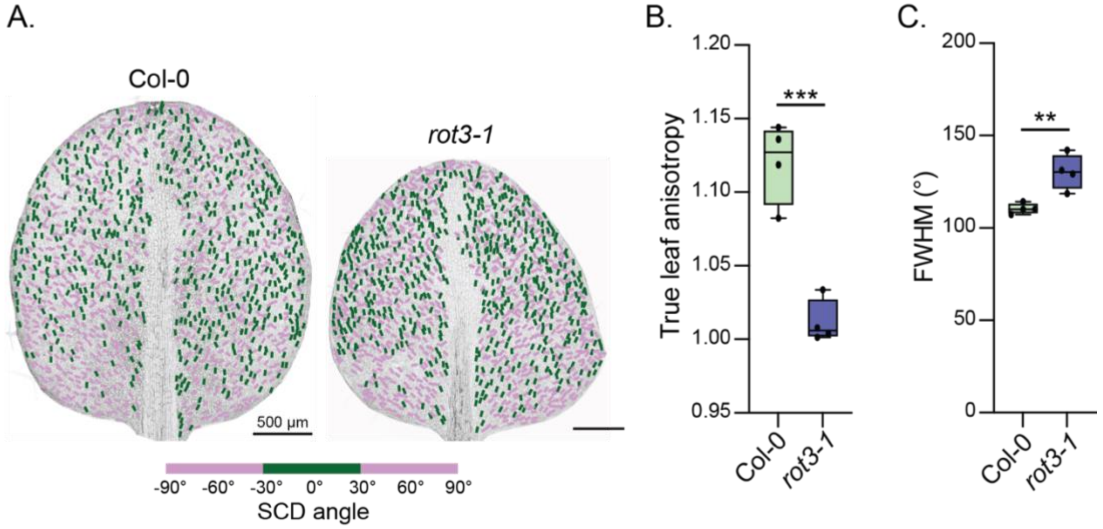

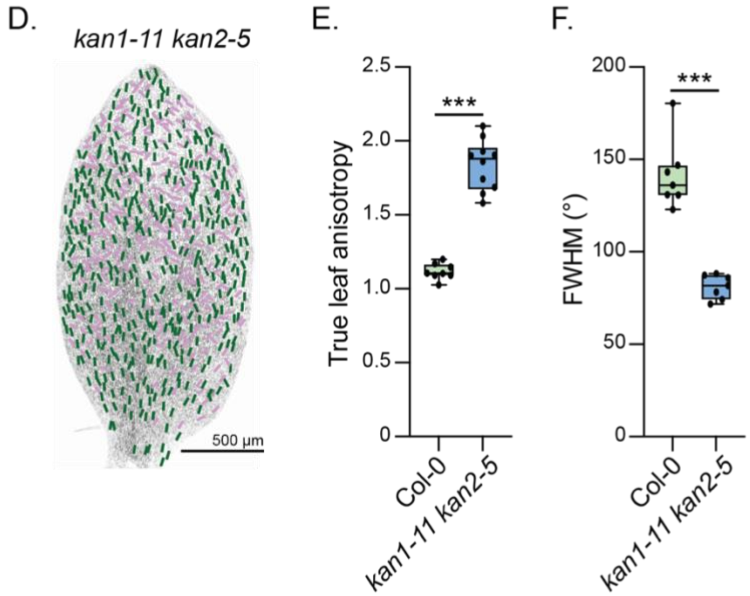

Fig. S5: Mutations that affect leaf shape also reorient the polarized SCD field.

A. Representative images of 10 dpg ML1p::mCherry-RCI2A-expressing Col-0 and *rot3-1* true leaves with color-coded SCDs. Scale bars – 500 µm.
B. Anisotropy of 10 dpg Col-0 and *rot3-1* true leaves. n = 4 true leaves each.
C. FWHM values for Col-0 and *rot3-1* true leaves in (B). n = 4 true leaves each.
D. Representative image of a 10 dpg ML1p::mCherry-RCI2A-expressing *kan1-11 kan2-5* true leaf with color-coded SCDs. Scale bar – 500 µm.
E. Anisotropy of 10 dpg Col-0 and *kan1-11 kan2-5* true leaves. n = 7 true leaves each.
F. FWHM values for Col-0 and *kan1-11 kan2-5* true leaves in (E). n = 7 true leaves each.

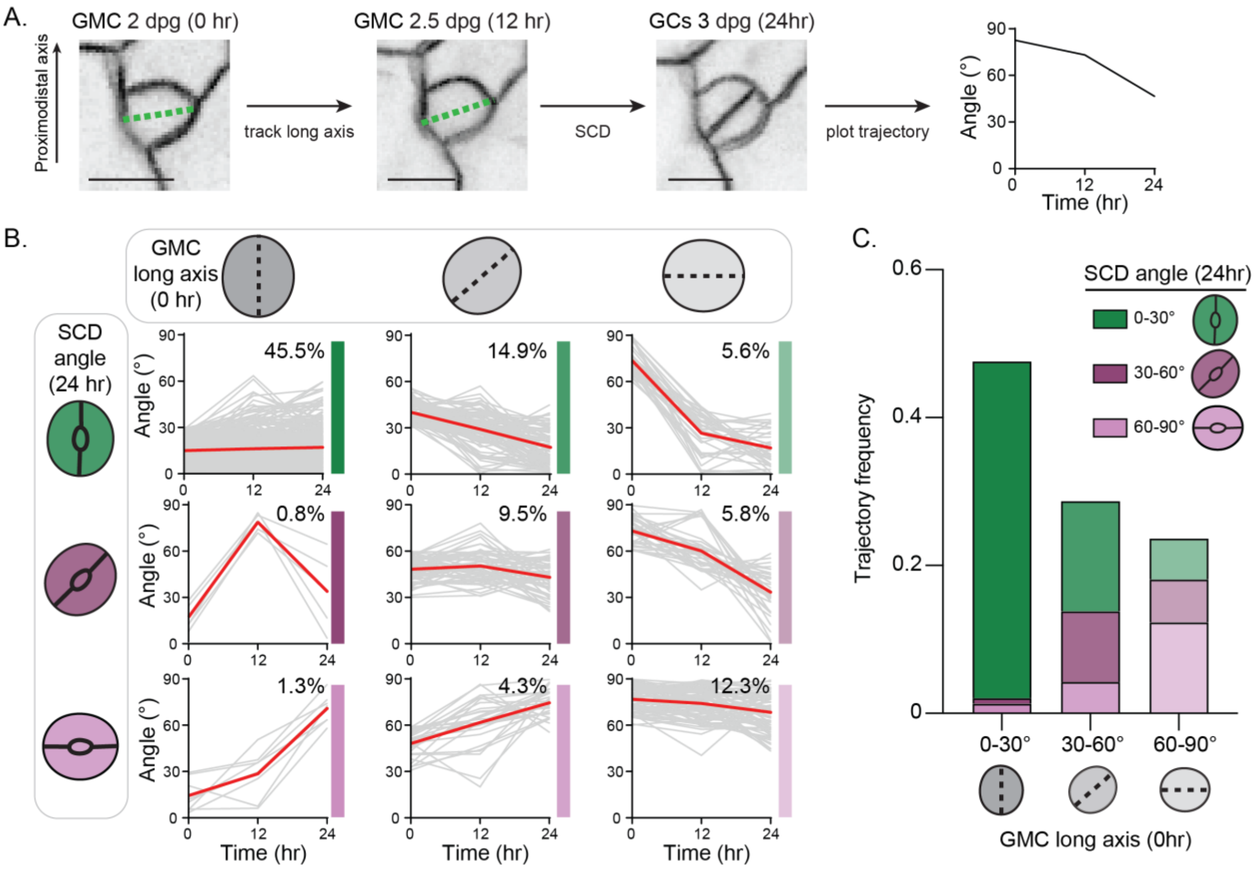

Fig. S6: Directional GMC expansion primes globally aligned SCDs.

A. Representative example of GMC tracking over 24 hours from 2 to 3 dpg. Scale bars – 5 μm.
B. Major patterns of directional cell expansion and SCD orientation following hierarchical clustering. The red lines indicate the average growth and SCD orientation for a given GMC class. The percent of total GMCs that fell within each class are shown in the upper right corners and the colored bars to the right correspond to the GMC behavioral categories shown in (C). n = 623 cells.
C. GMC behavioral categories based on hierarchical clustering.

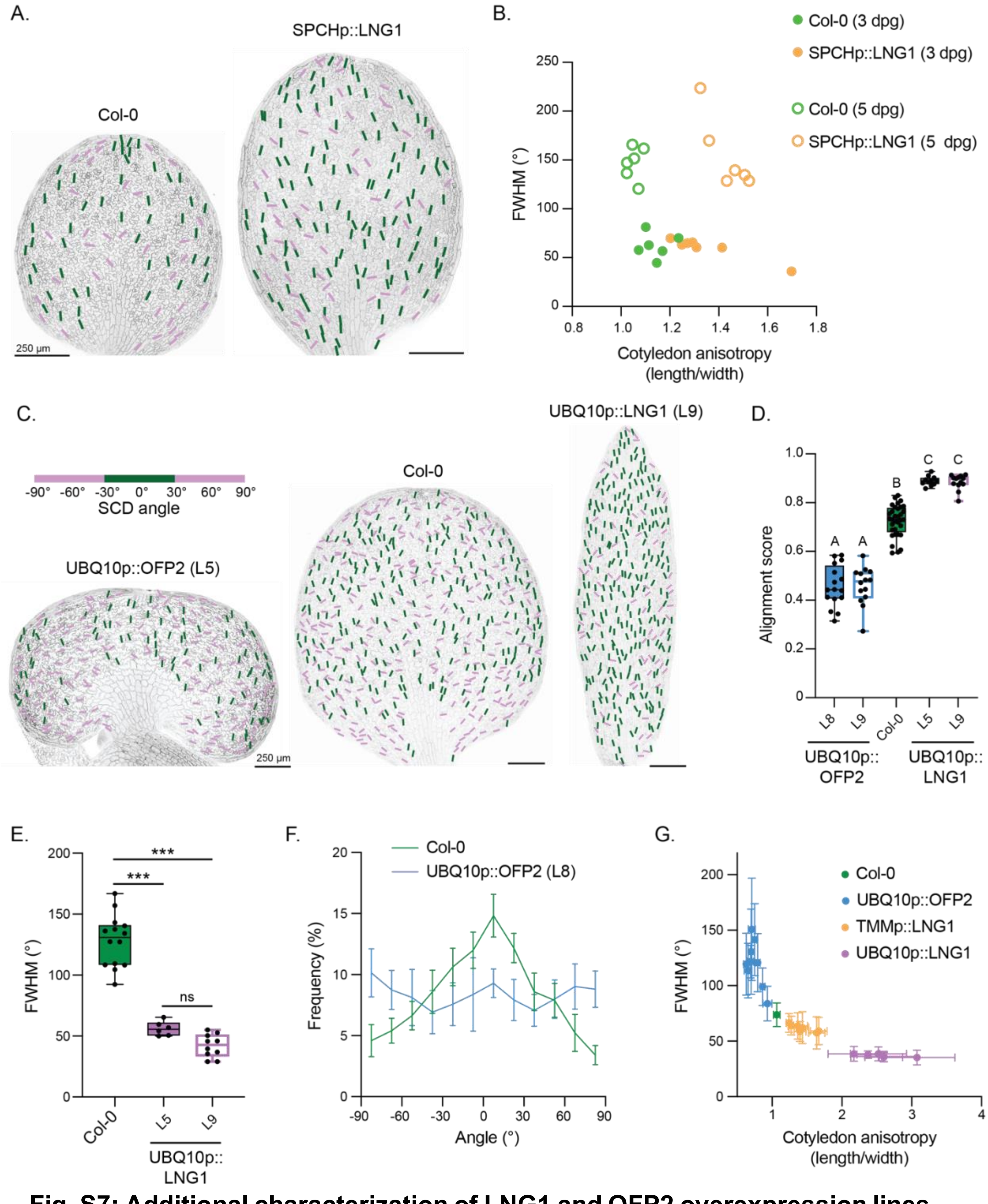

Fig. S7: Additional characterization of LNG1 and OFP2 overexpression lines.

A. Representative images of 3 dpg Col-0 and SPCHp::LNG1 cotyledons. Scale bars – 250 μm.

B. Comparison of FWHM values and the associated cotyledon anisotropy for 3 dpg and 5 dpg Col-0 and SPCHp::LNG1 cotyledons. n = 6 cotyledons each except for 3 dpg SPCHp::LNG1 (n=7).
C. Representative images of 5 dpg Col-0, UBQ10p::OFP2, and UBQ10p::LNG1 cotyledons. Scale bars – 250 μm.
D. Alignment scores for the indicated genotypes at 3 dpg. n = 33 (Col-0), 16 (UBQ10p::OFP2 L8), 14 (UBQ10p::OFP2 L9), 11 (UBQ10p::LNG1 L5) and 13 (UBQ10p::LNG1 L9) cotyledons.
E. FWHM values for the indicated genotypes at 5 dpg. n = 14 (Col-0), 6 (UBQ10p::LNG1 L5) and 10 (UBQ10p::LNG1 L9) cotyledons.
F. Frequency distributions for SCD angles in the indicated genotypes at 5 dpg. n = 10 seedlings each.
G. Comparison of FWHM values and the associated cotyledon anisotropy, where each point represents the mean value for independent transgenic lines of the indicated genotypes at 3 dpg. n = 9 (UBQ10p::OFP2), 7 (TMMp::LNG1), and 6 (UBQ10p::LNG1) independent transgenic lines. 8-18 cotyledons were analyzed for each line, with the exception of the paired Col-0 data (n = 55 cotyledons).

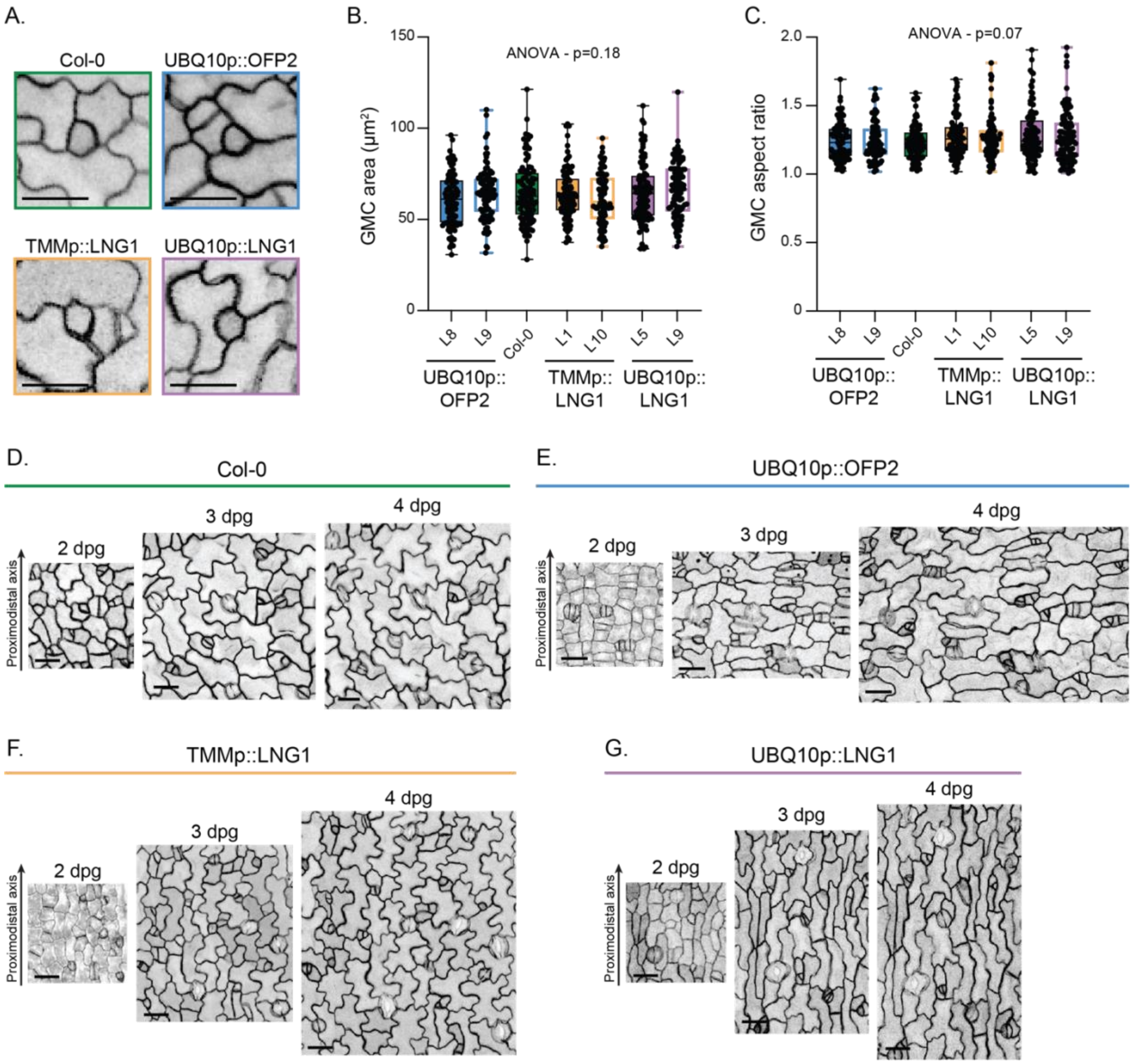

Fig. S8: Additional characterization of pavement cell growth and GMC morphology in LNG1 and OFP2 overexpression lines highlights non-cell autonomous role of cell expansion on SCD orientation.

A. Representative images of GMC morphology in the indicated genotypes (Col-0, UBQ10p::OFP2, TMMp::LNG1, and UBQ10p::LNG1). Scale bars – 20 μm.
B. GMC area in 3 dpg cotyledons of the indicated genotypes. n = 98 (Col-0), 98 (UBQ10p::OFP2 L8), 97 (UBQ10p::OFP2 L9), 98 (TMMp::LNG1 L1), 90 (TMMp::LNG1 L10), 102 (UBQ10p::LNG1 L5) and 96 (UBQ10p::LNG1 L9) GMCs.

C. GMC aspect ratio in 3 dpg cotyledons of the indicated genotypes (Col-0, UBQ10p::OFP2, TMMp::LNG1, and UBQ10p::LNG1). Same n values as (B).

D-G. Representative image series of directional pavement cell expansion in the indicated genotypes (Col-0, UBQ10p::OFP2, TMMp::LNG1, and UBQ10p::LNG1). Scale bars – 25 μm.

SI References

1. A. H. Roeder *et al.*, Variability in the control of cell division underlies sepal epidermal patterning in Arabidopsis thaliana. *PLoS Biol* **8**, e1000367 (2010).
2. M. H. Rowe, J. Dong, A. K. Weimer, D. C. Bergmann, A Plant-Specific Polarity Module Establishes Cell Fate Asymmetry in the Arabidopsis Stomatal Lineage. *bioRxiv*, 614636 (2019).
3. A. Muroyama, Y. Gong, K. S. Hartman, D. C. Bergmann, Cortical polarity ensures its own asymmetric inheritance in the stomatal lineage to pattern the leaf surface. *Science* **381**, 54-59 (2023).
4. J. Dong, C. A. MacAlister, D. C. Bergmann, BASL controls asymmetric cell division in *Arabidopsis*. *Cell* **137**, 1320-1330 (2009).
5. M. Yang, F. D. Sack, The too many mouths and four lips mutations affect stomatal production in Arabidopsis. *Plant Cell* **7**, 2227-2239 (1995).
6. L. B. Lai *et al.*, The Arabidopsis R2R3 MYB proteins FOUR LIPS and MYB88 restrict divisions late in the stomatal cell lineage. *Plant Cell* **17**, 2754-2767 (2005).
7. E. Schaefer *et al.*, The preprophase band of microtubules controls the robustness of division orientation in plants. *Science* **356**, 186-189 (2017).
8. D. Lin *et al.*, Rho GTPase signaling activates microtubule severing to promote microtubule ordering in Arabidopsis. *Curr Biol* **23**, 290-297 (2013).
9. G. Wu *et al.*, KANADI1 regulates adaxial-abaxial polarity in Arabidopsis by directly repressing the transcription of ASYMMETRIC LEAVES2. *Proc Natl Acad Sci U S A* **105**, 16392-16397 (2008).
10. G. T. Kim, H. Tsukaya, H. Uchimiya, The ROTUNDIFOLIA3 gene of Arabidopsis thaliana encodes a new member of the cytochrome P-450 family that is required for the regulated polar elongation of leaf cells. *Genes Dev* **12**, 2381-2391 (1998).
11. J. J. Petricka, N. K. Clay, T. M. Nelson, Vein patterning screens and the defectively organized tributaries mutants in Arabidopsis thaliana. *Plant J* **56**, 251-263 (2008).
12. L. E. Sieburth *et al.*, SCARFACE encodes an ARF-GAP that is required for normal auxin efflux and vein patterning in Arabidopsis. *Plant Cell* **18**, 1396-1411 (2006).
13. L. Fan *et al.*, Microtubules promote the non-cell autonomous action of microRNAs by inhibiting their cytoplasmic loading onto ARGONAUTE1 in Arabidopsis. *Dev Cell* **57**, 995-1008.e1005 (2022).
14. T. Bouquin, O. Mattsson, H. Naested, R. Foster, J. Mundy, The Arabidopsis lue1 mutant defines a katanin p60 ortholog involved in hormonal control of microtubule orientation during cell growth. *J Cell Sci* **116**, 791-801 (2003).
15. E. S. Wallner, L. Dolan, D. C. Bergmann, Arabidopsis stomatal lineage cells establish bipolarity and segregate differential signaling capacity to regulate stem cell potential. *Dev Cell* **58**, 1643-1656.e1645 (2023).

16. K. Ohashi-Ito, D. C. Bergmann, Arabidopsis FAMA controls the final proliferation/differentiation switch during stomatal development. *Plant Cell* **18**, 2493-2505 (2006).
17. M. J. Prigge *et al.*, Genetic analysis of the Arabidopsis TIR1/AFB auxin receptors reveals both overlapping and specialized functions. *Elife* **9** (2020).
18. X. He, S. Xu, L. Hong, 3D morphological analysis of Arabidopsis sepals. *Methods Cell Biol* **160**, 311-326 (2020).
19. A. Boudaoud *et al.*, FibrilTool, an ImageJ plug-in to quantify fibrillar structures in raw microscopy images. *Nat Protoc* **9**, 457-463 (2014).
20. P. Berens, CircStat: A MATLAB Toolbox for Circular Statistics. *Journal of Statistical Software* **31**, 1 - 21 (2009).
21. S. Marcotti *et al.*, A workflow for rapid unbiased quantification of fibrillar feature alignment in biological images. *Front Comput Sci* **3** (2021).
